# Pan-cancer analysis of the prognostic and immunological role of GJB2: a potential target for survival and immunotherapy

**DOI:** 10.3389/fonc.2023.1110207

**Published:** 2023-06-23

**Authors:** Yuting Jia, Bin Guo, Wenbin Zhang, Feng Wang, Yong Zhang, Quanmao Zhang, Erfeng Li

**Affiliations:** Department of Endoscopy Center, Shanxi Province Cancer Hospital, Taiyuan, China

**Keywords:** pan-cancer, prognosis, immune infiltration, TMB, MSI, ICP

## Abstract

**Background:**

GJB2 plays an essential role in the growth and progression of several cancers. However, asystematic pan-cancer analysis of GJB2 is lacking. Therefore, in this study, we performed a comprehensive pan-cancer analysis to determine the potential role of GJB2 in prognostic prediction and cancer immunotherapy response.

**Methods:**

The differential expression of GJB2 in the tumor and adjacent normal tissues of various cancer types was analyzed using the TIMER, GEPIA, and Sangerbox databases. GEPIA and Kaplan–Meier plotter databases were used to analyze the survival outcomes based on GJB2 expression levels in pan-cancer. Furthermore, the association of GJB2 expression with the immune checkpoint (ICP) genes, tumor mutational load (TMB), microsatellite instability (MSI), neoantigens, and tumor infiltration of immune cells was analyzed using *via* the Sangerbox database. The cBioPortal database was used to determine the characteristics of *GJB2* gene alterations in the cancer tissues. The STRING database was used to identify the GJB2-binding proteins. GEPIA database was used to identify the GJB2 co-expressed genes. DAVID was used to perform the functional enrichment analysis of gene ontology (GO) terms and KEGG pathways associated with GJB2. Finally, the mechanistic role of GJB2 in pancreatic adenocarcinoma (PAAD) was analyzed using the LinkedOmics database.

**Results:**

The *GJB2* gene was highly expressed in a variety of tumors. Furthermore, GJB2 expression levels showed significant positive or negative association with the survival outcomes in various cancers. GJB2 expression levels cor related with tumor mutational burden, microsatellite instability, neoantigens, and tumor infiltration of immune cells in multiple cancers. This suggested that GJB2 played a critical role in the tumor microenvironment. Functional enrichment analysis showed that the biological role of GJB2 in tumors included modulation of gap junction-mediated intercellular transport, regulation of cell communication by electrical coupling, ion transmembrane transport, autocrine signaling, apoptotic signaling pathway, NOD-like receptor signaling pathway, p53 signaling pathway, and PI3K-Akt signaling pathway.

**Conclusions:**

Our study demonstrated that GJB2 played a significant role in tumorigenesis and tumor immunity in multiple cancers. Furthermore, GJB2 is a potential prognostic biomarker and a promising therapeutic target in multiple types of cancers.

## Introduction

Cancer is a major public health issue and the leading cause of human deaths globally. The International Agency for Research on Cancer (IARC) reported 19.3 million new cancer cases and 10 million cancer deaths worldwide in 2020 (1). Tumorigenesis is a multi-faceted process that involves complex mechanisms regulating cancer cell proliferation and survival, tumor microenvironment, and tumor immune infiltration (2). Cancer patients experience high psychological stress and poor quality of life. Furthermore, patients with advanced stages of cancer are associated with worse prognosis than those diagnosed with cancer in the early stages. The main treatment options for cancer patients include surgery, chemotherapy, radiotherapy, targeted therapy, and immunotherapy.

In recent years, advances in molecular biology research have resulted in the emergence of molecular targeted therapies and immunotherapies based on the PD-1/PD-L1 inhibitors with improved outcomes. Most of the currently available targeted therapeutics act on single targets (genes or pathways). However, tumor pathogenetic mechanisms involve complex interactions between multiple factors. Therefore, multi-target drugs are required for better survival outcomes cancer patients. Furthermore, the efficacy of immunotherapy in cancer patients is limited by the complexity and diversity of the tumor microenvironment (TME) and the status of the tumor-infiltrated immune cells. These factors significantly influence the clinical outcomes of cancer patients that are treated with molecular targeted therapeutics and immunotherapies (3). Therefore, there is an urgent need to identify novel therapeutic targets and highly sensitive tumor biomarkers for cancer treatment.

Gap junction protein (GJB2), also known as connexin 26 (Cx26), is a member of the gap junction protein family, which is involved in the formation of the hemichannels and the gap junction channels. The opening of hemichannels allows the release of signaling molecules such as ATP and glutamate into the extracellular environment. The gap junction channels allow the exchange of ions and physiologically active molecules such as the second messengers between adjacent cells in direct contact through a process known as gap junctional intercellular communication (GJIC) (4). GJIC is closely related to cellular proliferation, differentiation, and apoptosis and its dysregulation is closely associated with oncogenesis (5). The aberrant expression of GJB2 causes dysregulation of GJIC in breast, colon, lung, and cervical cancers (6–8). Furthermore, connexins regulate cancer progression by modulating *via* the release of autocrine and/or paracrine signals into the extracellular environment through the hemichannels (9).

Teleki et al. (10) reported that GJB2 expression was reduced after chemotherapy in the breast cancer patients, thereby highlighting the association between GJB2 expression and the clinical response to chemotherapy. This also suggested that GJB2 was a promising anti-cancer drug target. However, to date the neoplastic role of GJB2 has been investigated in only a limited number of carcinomas. Moreover, pan-cancer analysis of the essential role of GJB2 has not been investigated. Therefore, we performed a comprehensive pan-cancer analysis of GJB2 expression levels in the tumor tissues and the adjacent normal tissues. Furthermore, we analyzed the prognostic value of GJB2 in pan-cancers and the association of GJB2 with the clinical pathological stages, immune checkpoint (ICP) genes, tumor mutation burden (TMB), microsatellite instability (MSI), and neoantigens. We also performed GJB2 gene co-expression analysis and gene set enrichment analysis in pan caner.

## Methods

### *GJB2* gene expression analysis

The TIMER2.0 database(http://timer.cistrome.org) was used to determine the differential expression levels of the *GJB2* gene in various tumor tissues and their corresponding adjacent normal tissues. Because paired tumor and normal tissues were not available for some tumors in the TIMER database, the “Expression analysis- Box Plots” module of the GEPIA database(http://gepia2.cancer-pku.cn/#analysis) was used to further confirm differential GJB2 expression in the pan-cancer tissues compared with the corresponding normal tissues by combining data from The Cancer Genome Atlas (TCGA) and the Geno type-Tissue Expression (GTEx) databases as controls. The TIMER 2.0 database does not contain data for the following tumor types: colon adenocarcinoma/rectum adenocarcinoma esophageal carcinoma (COADREAD), glioma (GBMLGG), pan-kidney cohort (KIPAN), and Stomach and Esophageal carcinoma (STES). Therefore, comprehensive analysis of GJB2 expression levels in the tumor and the corresponding normal tissues of the COADREAD, GBMLGG, KIPAN, and STES cohorts was performed using the Sangerbox database(http://Sangerbox.com/Tool). The Human Protein Atlas (HPA) database includes information regarding the spatial distribution and expression of various proteins in the human tissues and cells. We analyzed the GJB2 protein expression levels in various tumor tissues and their corresponding normal tissues based on the immunohistochemistry data in the HPA database.

### Prognosis and clinical phenotype analysis

The “Survival Analysis” module of GEPIA2 was used to determine the overall survival (OS) and the disease-free survival (DFS) rates based on the GJB2 expression levels (high or low) in patients with different types of tumors from the TCGA database (11). We also analyzed the association between GJB2 expression and overall survival (OS) as well as relapse-free survival (RFS) in pan-cancer using the Kaplan–Meier Plotter database. The correlations between GJB2 expression levels and the clinicopathological parameters such as clinical stages, grades, sex, and age in pan cancer were analyzed using the Sangerbox database. The data for clinical stages, grades, and gender were shown as box plots, whereas the correlation between GJB2 expression and age was shown as bubble plots. *P*< 0.05 was considered as statistically significant.

### Analysis of *GJB2* gene alterations

The cBioPortal database was used to analyze the *GJB2* gene mutation frequency, types, and copy number alterations (CNA) in the pan-cancer tissues (12). The “Mutations” module in the cBioPortal database was used to display the mutation site information of GJB2 and the corresponding position in the 3D protein structure. We also explored the mutation count of GJB2 in pan-cancer.

### Correlation analysis between GJB2 expression levels and tumor immunity markers

The Sangerbox database was used to analyze the relationship between GJB2 expression levels and critical tumor immunity biomarkers such as immune checkpoint (ICP) genes (including inhibitory ICPs and stimulatory ICPs), tumor mutational load (TMB), microsatellite instability (MSI), and neoantigens in theTME (13). *P* value < 0.05 was considered as statistically significant.

### Analysis of tumor immune infiltration

The Sanger Box database was used to download the standardized pan-cancer dataset (TCGA Pan-Cancer data from the UCSC database). GJB2 gene expression data was extracted for each sample and transformed using log_2_(X + 0.001). Furthermore, GJB2 expression profile of each tumor was extracted separately and mapped to the GeneSymbol. The ESTIMATE R package (version: 1.0.13) https://bioinformatics.mdanderson.org/public-software/estimate/) was used to calculate the stromal, immune, and ESTIMATE scores for all the patients with different types of tumors. Furthermore, the TIMER algorithm and the deconvo_EPIC, IPS, MCPcounter, CIBERSORT, xCell, and QUANTISEQ algorithms in the IOBR R package (version 0.99.9) (https://www.ncbi.nlm.nih.gov/pmc/articles/PMC8283787/) were used to analyze the relationship between GJB2 gene expression levels and tumor immune cell infiltration in various tumors.

### Functional enrichment analysis

The STRING database was used to construct a protein–protein interaction (PPI) network of the predicted GJB2- binding proteins. The STRING database was searched to identify the potential GJB2 binding proteins. Then, the top 50 GJB2-related target genes were identified by analyzing all the different cancer datasets in the TCGA database (tumor and normal tissues) using the “Similar Gene Detection” module of GEPIA2. Pearson correlation analysis was then performed between GJB2 and the 50 GJB2-related target genes u sing the “Correlation Analysis” module of GEPIA2. The heat map data of the selected genes was generated using the “Gene Corr” module of TIMER2. The results of the two datasets were intersected using a Venn diagram to to obtain the common genes of the two datasets. The genes in the two data sets were combined and the Kyoto Encyclopedia of Genes and Genomes (KEGG) pathway and Gene Ontology (GO) enrichment analyses was performed. The gene list was uploaded to DAVID (Database for Annotation, Visualization, and Integrated Discovery) and the functional annotation map was generated. Then, the results were visualized using the Sangerbox tool.

### GJB2 gene co-expression network analysis

Finally, we further validated the enriched biological functions and pathways related to GJB2 in PAAD using the LinkedOmics database. The LinkedOmics database was used to determine the correlation coefficients of genes that co-express with GJB2. The results were displayed as heatmaps and volcano plots. Then, we investigated Gene Ontology Biological processes (GO_BP) and the KEGG pathways of GJB2 and its coexpression genes utilizing Gene Set Enrichment Analysis (GSEA).

## Results

### GJB2 is differentially expressed in several cancers

**Figure 1:**
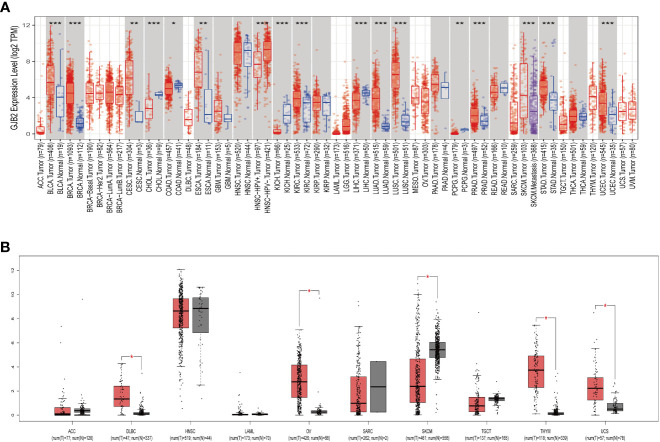
*GJB2* gene expression in different cancers. **(A)** GJB2 expression levels in the tumor and normal tissues from the TCGA pan-cancer datasets using the TIMER2.0 database. **(B)** GJB2 expression levels in the paired tumor/normal samples of the pan-cancer datasets from the TCGA and GTEx databases. *P < 0.05, **P < 0.005, ***P < 0.001.

The TIMER2.0 database analysis showed that GJB2 expression was significantly higher in BLCA, BRCA, CESC, ESCA, KIRC, LUAD, LUSC, PRAD, STAD and UCEC but significantly reduced in CHOL, COAD, KICH, LIHC, and PCPG (**Figure 1A**). Since the TIMER 2.0 database lacked normal controls for ACC, DLBC, HNSC, LAML, OV, SARC, SKCM, TGCT, THYM, and UCS tumors, we performed a supplemental analysis using the GEPIA database, which includes data from the TCGA and GTEx databases. The analysis results showed that GJB2 expression was significantly higher in DLBC, OV, THYM, and UCS but was reduced in SKCM (**Figure 1B**). Then, we performed comprehensive analysis of GJB2 expression in 33 different cancers and adjacent normal tissues using the Sangerbox database and found that GJB2 was overexpressed in GBM, UCEC, BRCA, CESC, LUAD, ESCA, STES, KIRP, KIPAN, COAD, COADREAD, PRAD, STAD, KIRC, LUSC, BLCA, THCA, OV, PAAD, UCS, and ALL but downregulated in LGG, LIHC, SKCM, TGCT, LAML, PCPG, ACC, KICH, and CHOL (**Supplementary Figure 1**). The results from both Sangerbox and TIMER2.0 databases showed consistent trends in the GJB2 expression profiles in various tumors.

**Figure 2:**
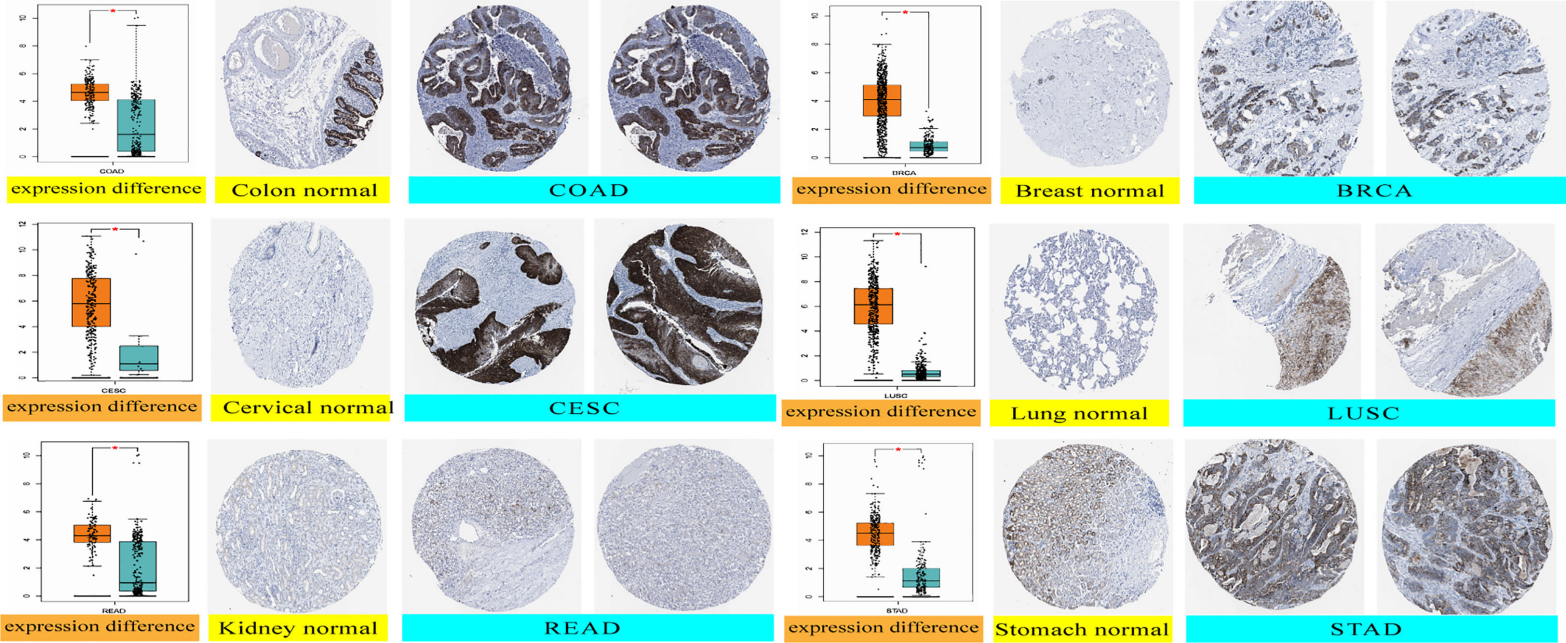
GJB2 protein expression levels in the normal and tumor tissues of the colon, cervical, kidney, breast, lung, and stomach cancer datasets from the HPA database. *P < 0.05.

We analyzed the immunohistochemistry data in the HPA database to determine the expression levels of the GJB2 protein level in partial tumors. GJB2 protein expression levels were higher in the COAD, BRCA, CESC, LUSC, READ, and STAD tissues compared to the corresponding normal tissues (**Figure 2**).

### GJB2 expression correlates with survival outcomes in several cancers

**Figure 3:**
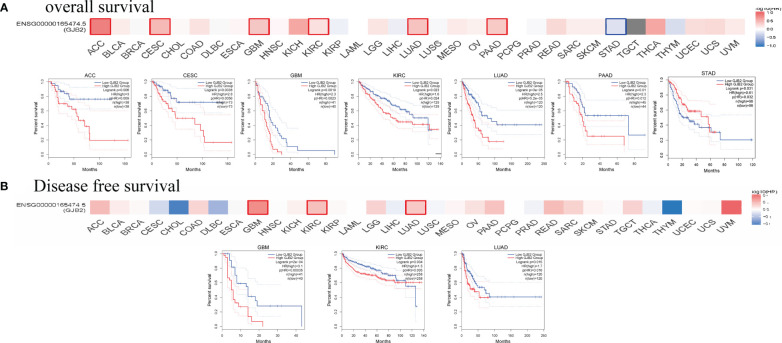
Survival outcomes of cancer patients based on high and low GJB2 expression in the pan-cancer datasets using the GEPIA2 tool. **(A)** The analysis of overall survival (OS) based on the level of *GJB2* gene expression in the TCGA pan-cancer datasets. **(B)** The analysis of disease-specific survival (DSS) based on the level of *GJB2* gene expression in the TCGA pan-cancer datasets.

The cancer patients were classified into high and low expression groups based on the GJB2 expression levels and the survival outcomes were determined based on the GJB2 expression levels in various tumors to determine the prognostic value of GJB2. The GEPIA database analysis showed that higher GJB2 expression was associated with worse OS outcomes in patients with ACC, CESC, GBM, KIRC, LUAD, and PAAD (all *P* < 0.05). However, high GJB2 gene expression was associated with better OS outcomes in patients with STAD (*P* < 0.05). Furthermore, high GJB2 expression correlated with poorer DFS in patients with GBM, KIRC, and LUAD (all *P* < 0.05) (**Figures 3A, B**).

**Figure 4:**
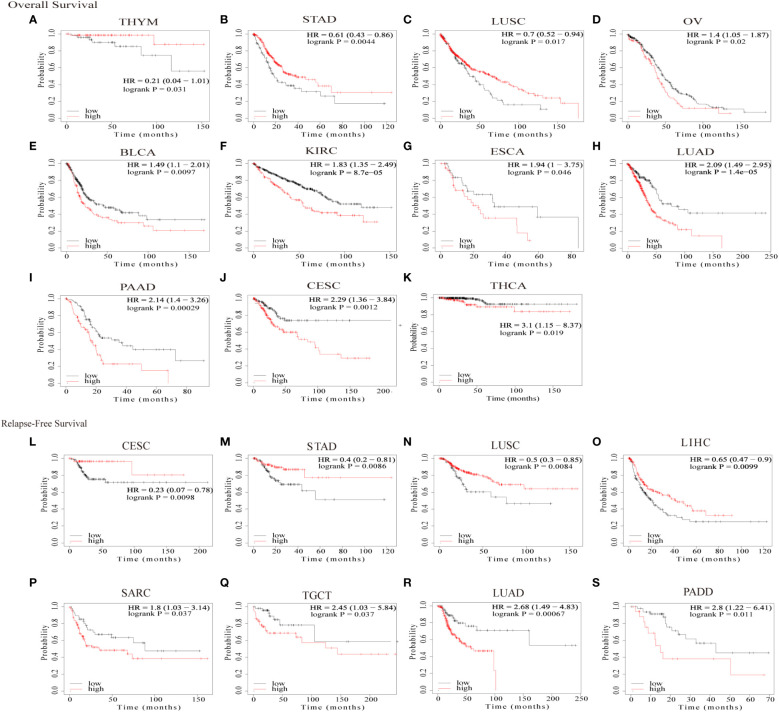
Kaplan–Meier survival curve analysis of pan-cancers based on *GJB2* gene expression levels. **(A–K)** The analysis of overall survival (OS) based on the level of *GJB2* gene expression in the TCGA pan-cancer datasets; **(L–S)** The analysis of relapse-free survival (RFS) based on the level of *GJB2* gene expression in the TCGA pan-cancer datasets.

We then assessed the prognostic value of GJB2 in pan-cancer using the Kaplan–Meier plotter database. Kaplan–Meier survival curves showed that GJB2 were associated with poorer OS outcomes in patients with OV, BLCA, KIRC, ESCA, LUAD, PAAD, CESC, and THCA (all *P*<0.05), but was associated with better OS outcomes in patients with THYM, STAD, and LUSC (all *P*<0.05) (**Figures 4A–K**). Kaplan–Meier survival curve analysis demonstrated that high GJB2 expression levels were associated with poor RFS outcomes in patients with PAAD, LUAD, TGCT, and SARC, but were associated with better RFS outcomes in patients with CESC, STAD, LUSC, and LIHC (**Figures 4L–S**). These data showed that the prognostic outcomes based on the expression levels of GJB2 varied between different tumors. In conclusion, these data demonstrated that GJB2 was a potential prognostic biomarker in multiple types of cancer.

**Figure 5:**
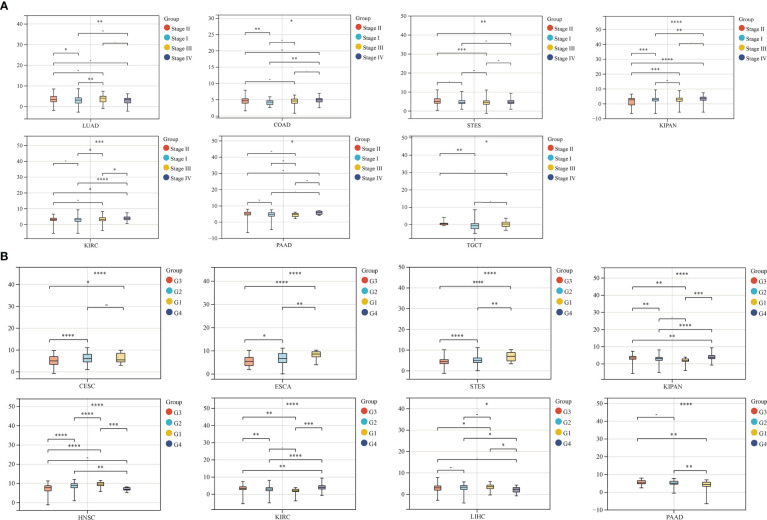
Correlation analysis of GJB2 expression with clinical stages and grades of various tumors. **(A)** GJB2 expression levels show significant correlation with different clinical stages in patients witxh LUAD, COAD, STES, KIPAN, KIRC, PAAD, and TGCT. **(B)** GJB2 expression levels demonstrate significant association with the grade of CESC, ESCA, STES, KIPAN, HNSC, KIRC, LIHC, and PAAD. ^∗^ *P* < 0.05, ^∗∗^ *P* < 0.01, ^∗∗∗^ *P* < 0.001, ****P < 0.0001, and - *P*≥0.05.

**Figure 6:**
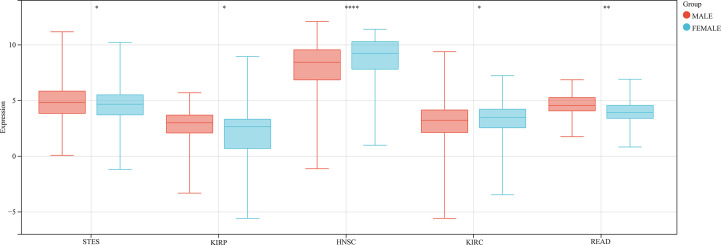
Correlation analysis between GJB2 expression levels and se in the pan-cancer datasets. As shown, GJB2 expression significantly correlates with sex in STES, KIRP, HNSC, KIRC, and READ. *P < 0.05, **P <0.01, and ****P < 0.0001.

**Figure 7:**
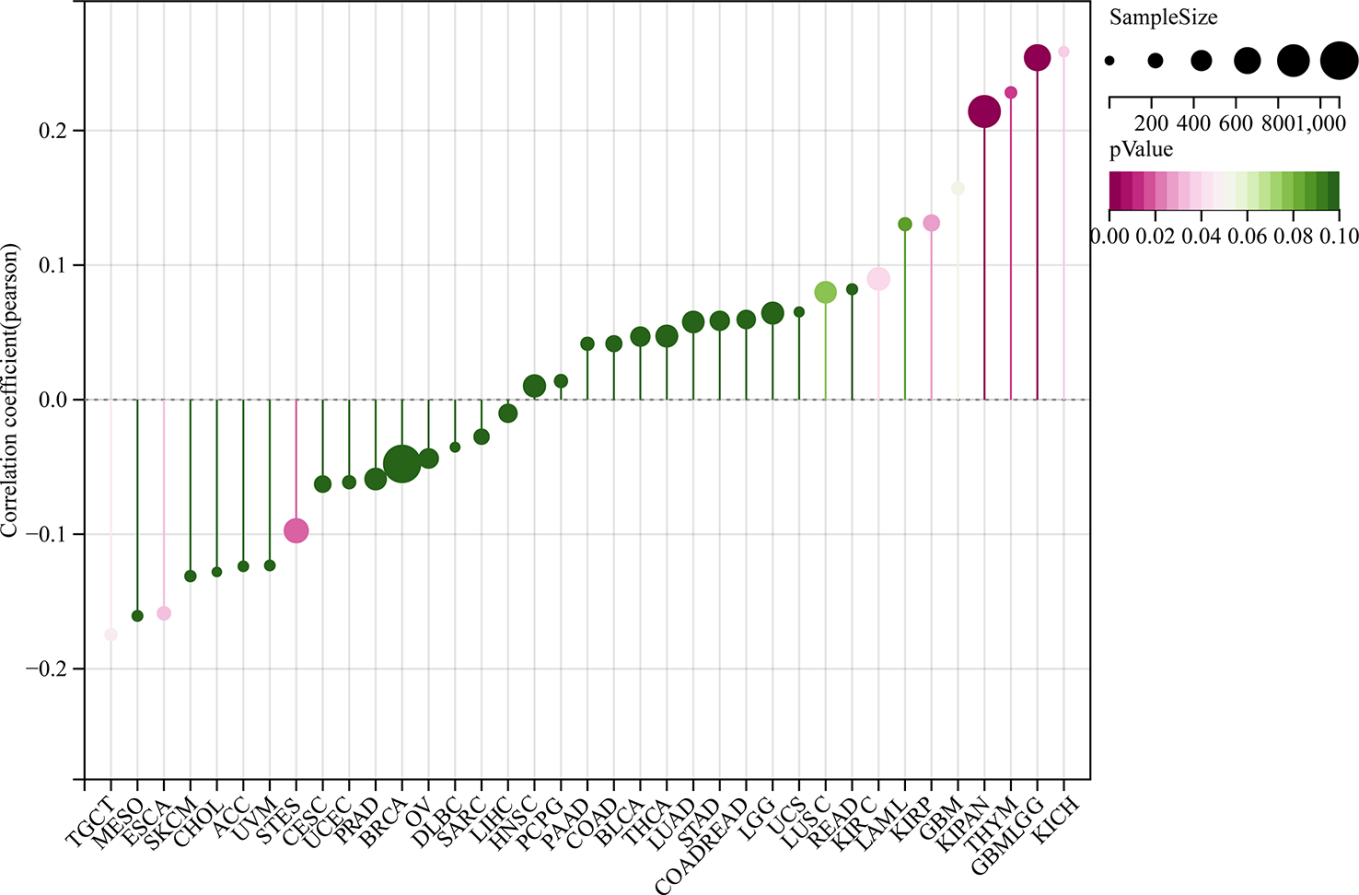
Correlation analysis between GJB2 expression levels and age in the pan-cancer datasets. As shown, GJB2 expression positively correlates with age in GBMLGG, KIRP, KIPAN, KIRC, THYM, and KICH, and negatively correlates with age in ESCA, STES, and TGCT. Note: The different color codes indicate the size of different p-values; the direction and length of the vertical axis indicates positive or negative correlation between GJB2 and age; Cor represents correlation efficient; size of the circles indicates sample size.

We also analyzed the correlation between GJB2 expression levels and clinicopathological parameters of cancer such as clinical stages, grades, gender, and age using the Sangerbox database. GJB2 expression levels showed significant correlation with the clinical stages in LUAD, COAD, STES, KIPAN, KIRC, PAAD, and TGCT (all *P*<0.05) (**Figure 5A**). Furthermore, GJB2 expression levels showed significant correlation with the tumor grades in CESC, ESCA, STES, KIPAN, HNSC, KIRC, LIHC, and PAAD (**Figure 5B**) (all *P*<0.05). GJB2 expression levels showed significant correlation with gender in STES, KIRP, HNSC, KIRC, and READ (all *P*<0.05) (**Figure 6**). Furthermore, GJB2 expression levels showed significant positive correlation with age in GBMLGG, KIRP, KIPAN, KIRC, THYM, and KICH (all *P*<0.05) and significant negative association with age in ESCA, STES, and TGCT (all *P*<0.05) (**Figure 7**).

### Analysis of *GJB2* gene alterations in pan-cancer datasets

**Figure 8:**
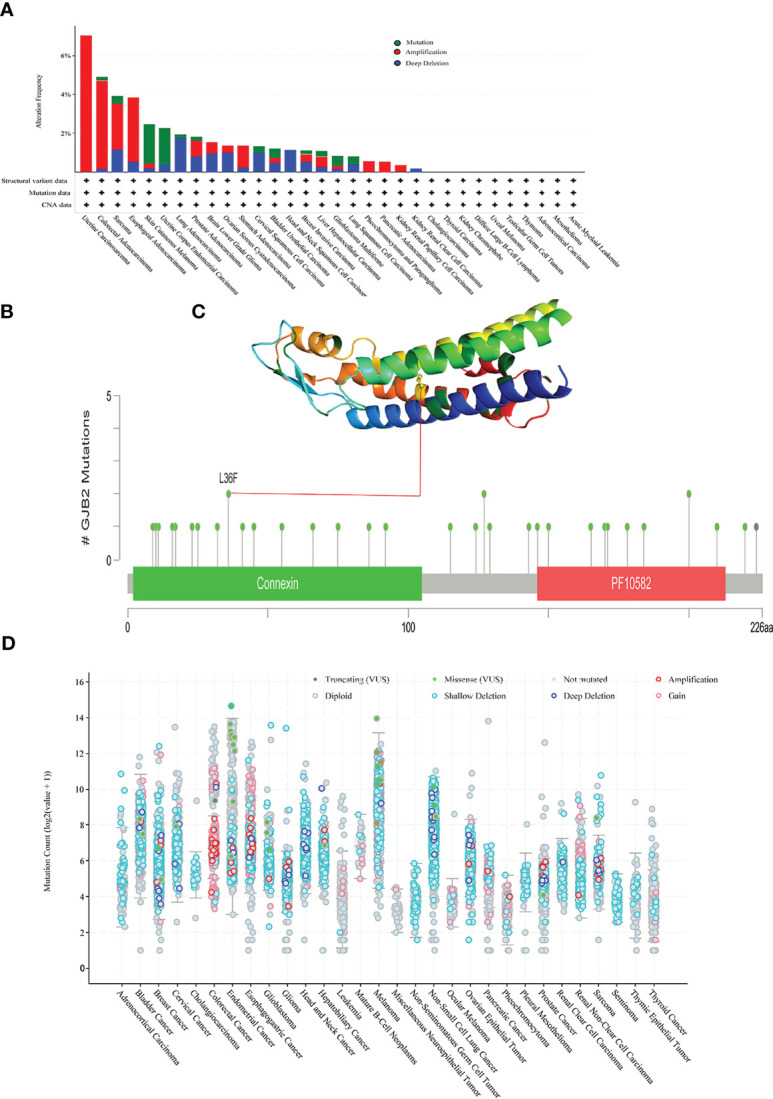
Mutational features of the *GJB2* gene in various cancers. **(A)** The mutational frequency and mutation type of the *GJB2* gene in various cancers. **(B)** The mutation counts of the *GJB2* gene in various cancer types from the TCGA database. The mutational types are represented by differentially colored. **(C)** 3D structure of GJB2 with L36F, which represents the site with the highest mutational frequency among all cancers. **(D)** General mutation count of the *GJB2* gene in various cancer types from the TCGA database.

We used the cBioPortal database to analyze mutations in the *GJB2* gene in pan-cancer datasets. *GJB2* gene mutation frequency was highest in SKCM. Furthermore, all the alterations in the *GJB2* gene were copy number amplifications (CNA) in UCS, PCPG, PAAD, and KIRP. In HNSC and KIRC, deep deletions were observed in the *GJB2* gene (**Figure 8A**). The specific *GJB2* gene mutations including mutation types, mutation sites, and the corresponding number of cases were listed for all the cancer types. Missense mutations were the main type of GJB2 genetic alterations, and L36F mutation was identified in two SKCM patients (**Figure 8B**). L36F site in the 3D structure of the GJB2 protein is shown in **Figure 8C**. The *GJB2* mutation counts in pan-cancer datasets are shown in **Figure 8D**.

### GJB2 expression correlates with ICP genes, TMB, MSI, and neoantigens in pan-cancer datasets

**Figure 9:**
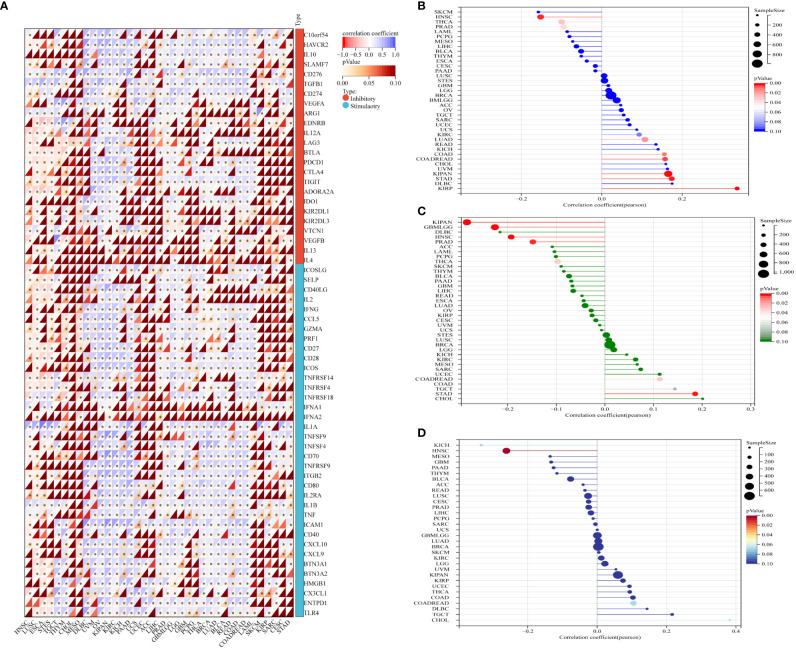
Association between GJB2 expression and tumor immunity biomarkers. **(A)** The relationship between GJB2 expression and immune checkpoint (ICP) genes [inhibitory (24) and stimulatory (36)] in pan-cancer. Each small rectangular module represents co-expression of immune-related genes and GJB2 in various cancers; color in the upper left corner represents the correlation coefficient (Cor); the asterisk and color in the lower right corner represents the P value. **(B–D)** The relationship of GJB2 expression levels with **(B)** TMB, **(C)** MSI, and **(D)** neoantigens. The different colors represent the P-value. The horizontal axis represents the positive/negative correlation, including the magnitude of the correlation between GJB2 expression and age in pan-cancer. **(B)** GJB2 expression shows significant positive association with TMB in LUAD, COAD, COADREAD, KIRP, KIPAN, and STAD; GJB2 expression shows significant negative association with TMB in PRAD, HNSC, and THCA. **(C)** GJB2 expression shows positive correlation with MSI in COADREAD and STAD; GJB2 expression shows negative correlation with MSI in GBMLGG, KIPAN, PRAD, HNSC, and THCA. **(D)** GJB2 expression shows positive correlation with neoantigens in HNSC. ^∗^ *P* < 0.05, ^∗∗^ *P* < 0.01, and ^∗∗∗^ *P* < 0.001.

Immune checkpoint (ICP) genes play a significant role in the tumor infiltration of immune cells and immunotherapy responses (14). Immune checkpoint proteins are key regulators of immunity by activating orsuppressing critical immune regulatory signaling pathways. Thus, ICP proteins are critical for the maintenance of self-tolerance and immune responses. Furthermore, immune checkpoint-related genes play a key role in the immune escape mechanisms of tumors. Therefore, we analyzed the correlation between expression levels of GJB2 and the ICP genes to determine the role of GJB2 in immunotherapy. GJB2 expression levels were associated with 60 ICP genes in cancer types such as HNSC, LUSC, ESCA, STES, OV, KIPAN, KIRC, KICH, PAAD, THCA, BRCA, LUAD, READ, COAD, and COADREAD. Additionally, GJB2 expression showed positive correlation with the expression of several immune-related genes in GBM, THCA, BRCA, LUAD, BLCA, READ, COAD, LAML, SKCM, and KIRP. However, GJB2 expression showed negative association with the expression of several immune-related genes in HNSC, LUSC, ESCA and STES. These data demonstrated that GJB2 expression correlated with immune-related genes in most tumors (**Figure 9A**). Therefore, GJB2 could be a promising target for tumor therapy.

Previous studies have shown that TMB, MSI, and neoantigens are significantly associated with the tumor immunotherapy responses and are used as predictive biomarkers of the immunotherapy response in the cancer patients (15–17). Therefore, we analyzed the relationship between GJB2 expression levels and the status of TMB, MSI, and neoantigens (NEO) in all the tumors from the TCGA database. GJB2 expression levels showed positive correlation with TMD in patients with LUAD, COAD, COADREAD, KIPAN, KIRP, and STAD, but demonstrated negative correlation with TMD in patients with PRAD, HNSC, and THCA (all *P*<0.05) (**Figure 9B**). GJB2 expression levels showed positive correlation with MSI in patients with COADREAD and STAD, but showed negative correlation with MSI in patients with GBMLGG, KIPAN PRAD, HNSC, and THCA (all *P <*0.05) (**Figure 9C**). Furthermore, GJB2 expression levels showed negative correlation with neoantigens in patients with HNSC (*P <*0.001) (**Figure 9D**). These results suggested that GJB2 expression influenced the status of antitumor immunity by regulating the TME, including mechanisms related to tumor immunity.

### GJB2 expression correlates with tumor immune infiltration in pan-cancer datasets

**Figure 10:**
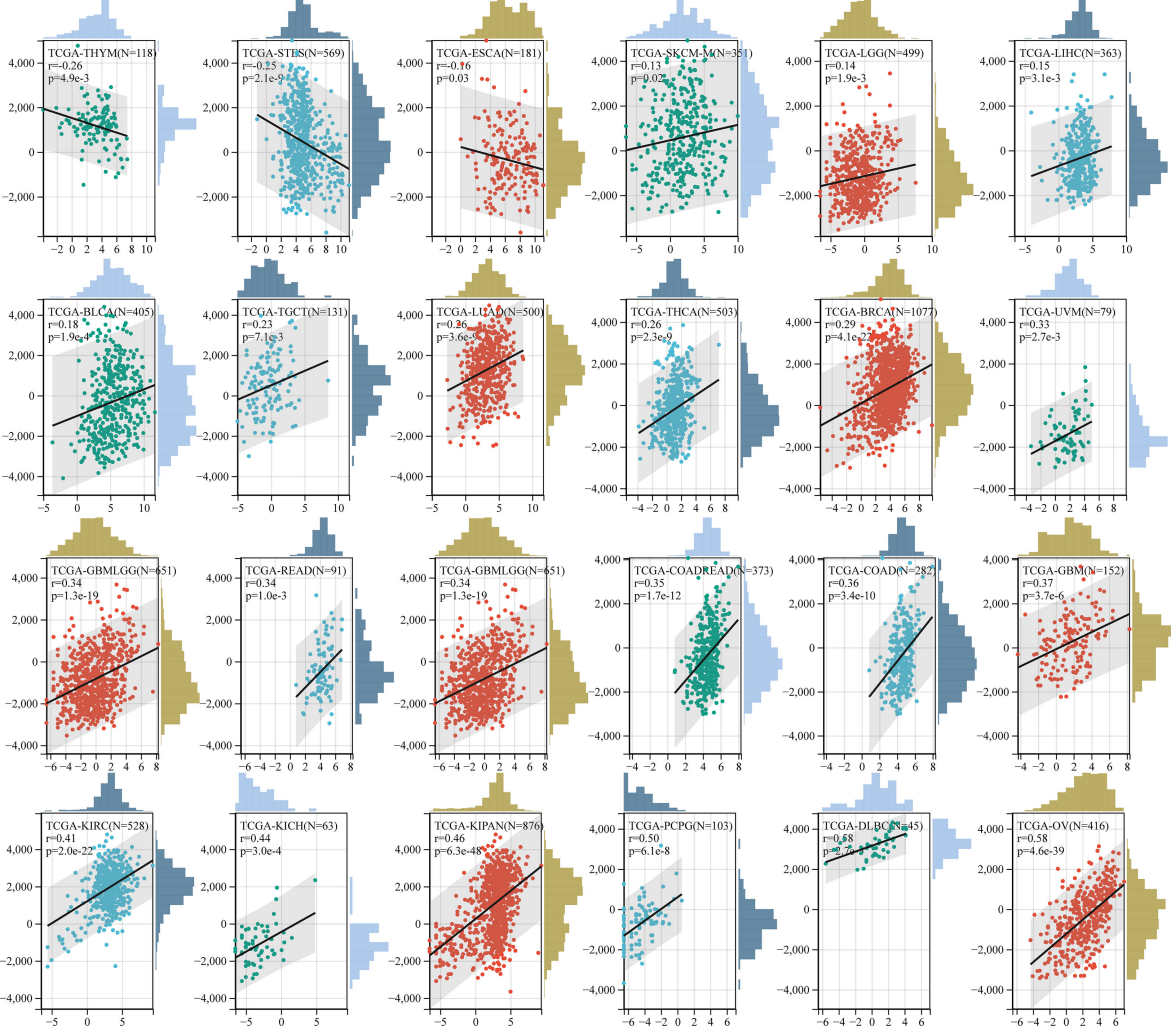
Correlation analysis between GJB2 expression and ESTIMATE scores in pan-cancer using the ESTIMATE algorithm.

The relationship between GJB2 expression levels and the tumor immune infiltration status in pan-cancer was analyzed by estimating the immune scores, stromal scores, and the ESTIMATE scores. In most cancers, GJB2 expression levels showed positive correlation with the stromal scores (**Supplementary Figure 3**) and the immune scores (**Supplementary Figure 4**). However, GJB2 expression levels showed negative correlation with the immune scores in ESCA, LUSC, THYM and MESO. Furthermore, in most cancer types, GJB2 expression levels showed positive correlation with the ESTIMATE scores (**Figure 10**).

**Figure 11:**
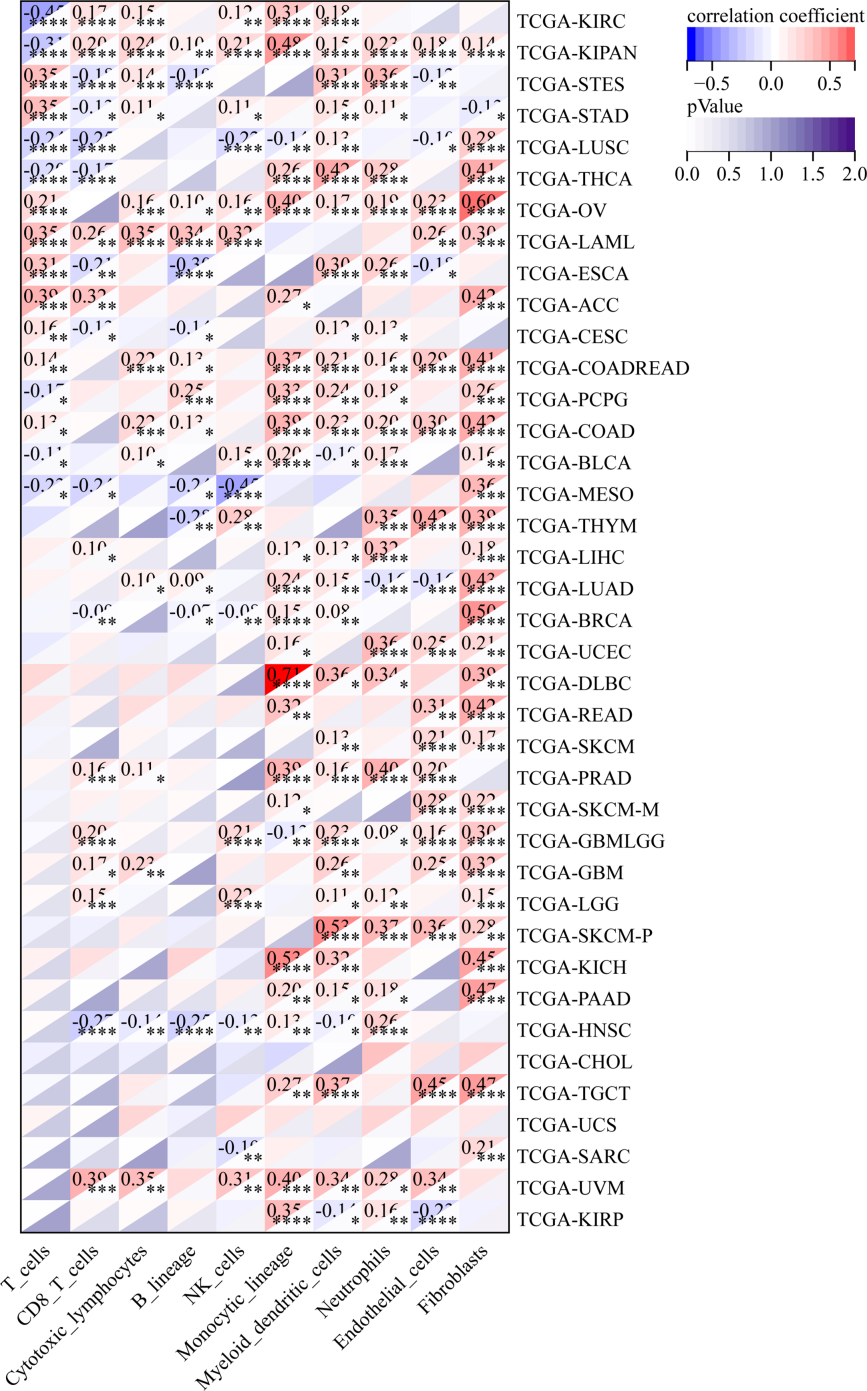
MCPCOUNTER analysis results show significant correlation between GJB2 expression levels and the infiltration levels of various immune cells. *P <0.05, **P < 0.01, ***P < 0.001, and ****P < 0.0001.

**Figure 12:**
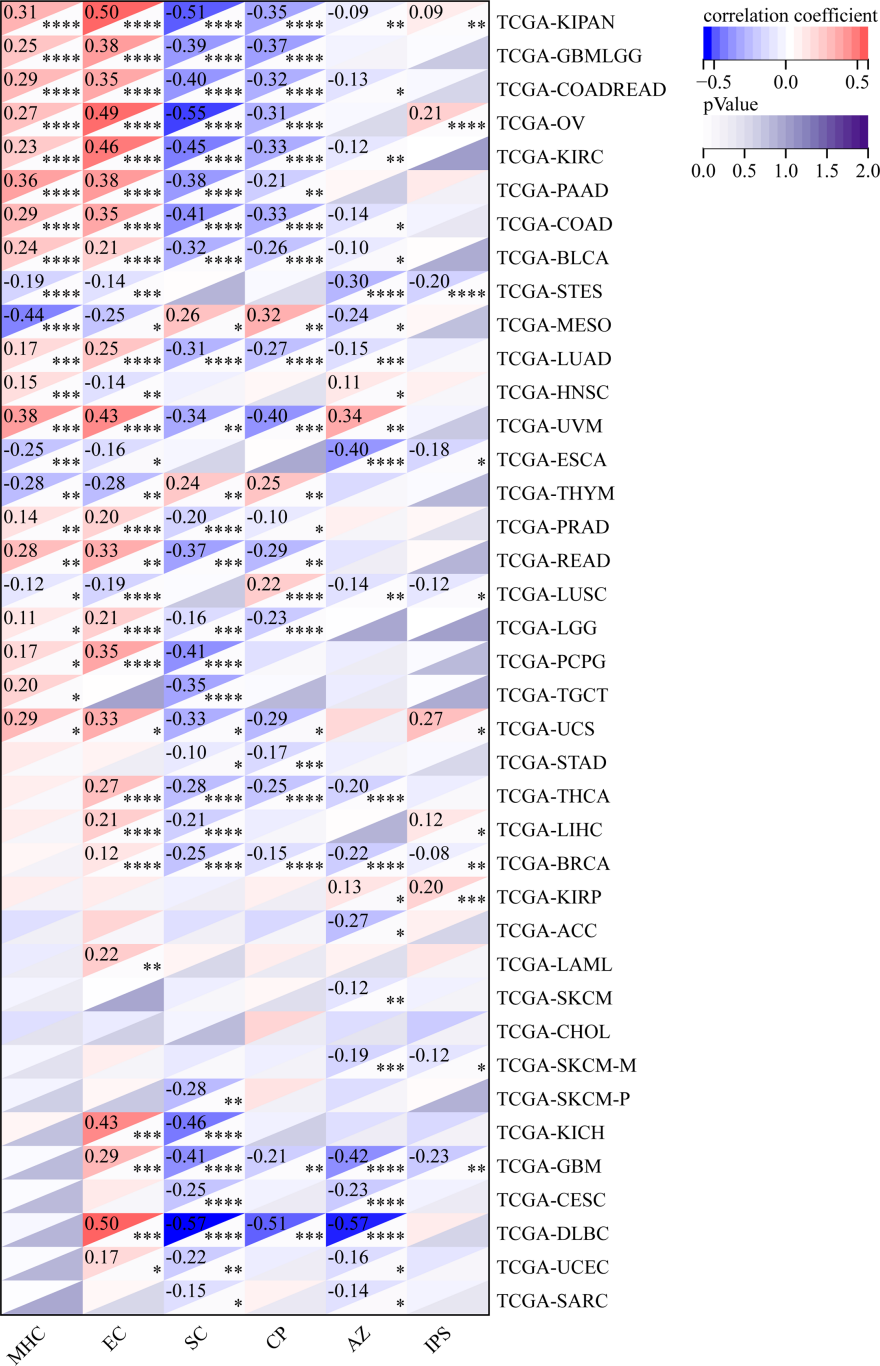
IPS analysis results show significant correlation between GJB2 expression levels and the infiltration levels of various immune cells. *P < 0.05, **P <0.01, ***P < 0.001, and ****P < 0.0001.

**Figure 13:**
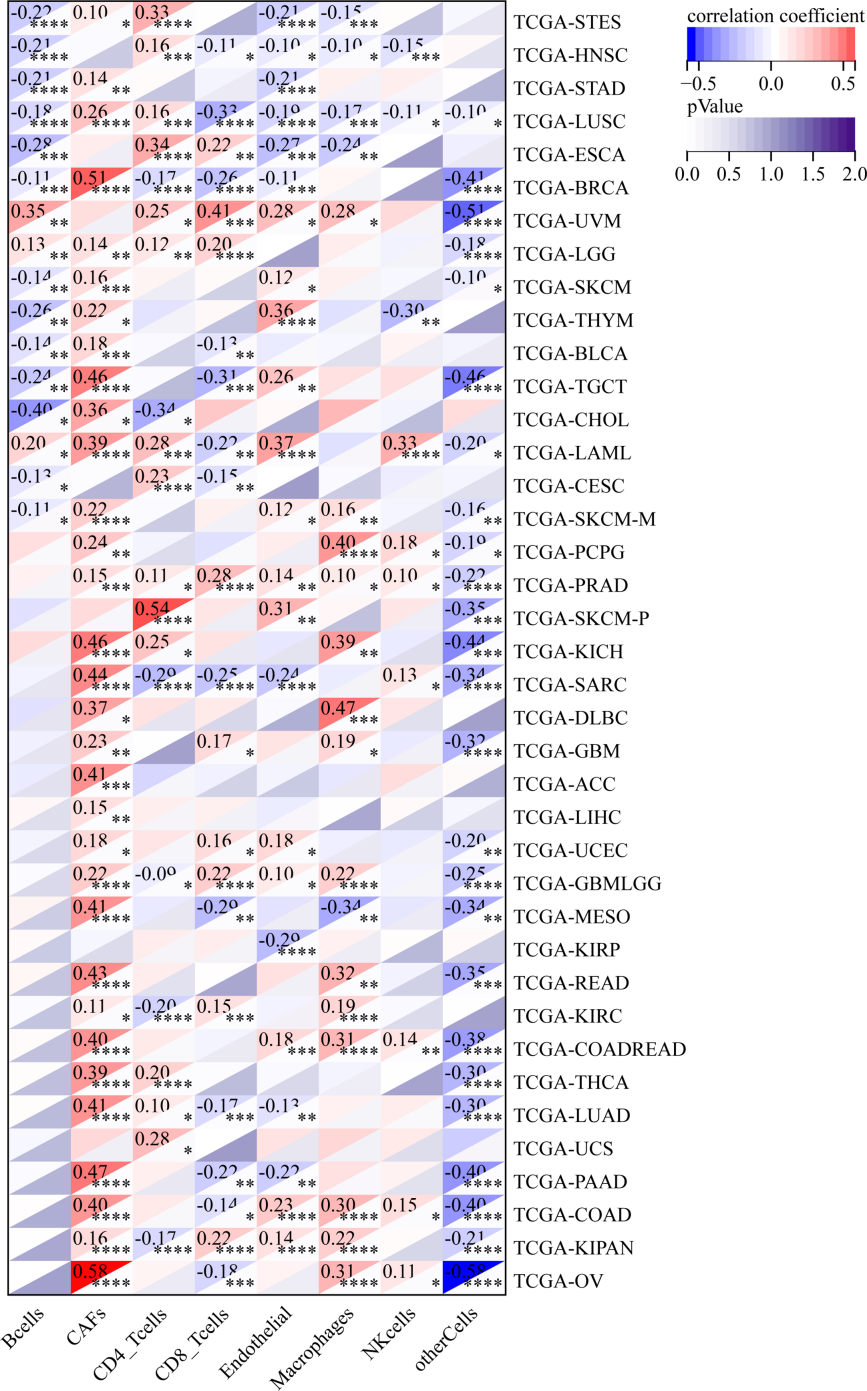
EPIC analysis results show significant correlation between the GJB2 expression levels and the infiltration levels of various immune cells. *P < 0.05, **P < 0.01, ***P < 0.001, and ****P < 0.0001.

We also analyzed the relationship between the infiltration levels of immune cells and GJB2 expression levels in different types of tumors in the TCGA database using the CIBERSORT, QUANTISEQ, MCPCOUNTER, IPS, TIMER, EPIC, and the XCELL algorithms (**Figure 11**–**13**; **Supplementary Figures 2, 5–7**). TIMER database analysis showed positive correlation between GJB2 expression levels and the infiltration of B cells, T cell CD4^+^ T cells, CD8^+^ T cells, neutrophils, macrophages, and dendritic cells (DCs) in THCA, PRAD and KIRC. Furthermore, tumor infiltration levels of neutrophils, macrophages and DCs showed significant positive correlation with the GJB2 expression levels in several tumors, especially CHOL, KICH, and COAD. Moreover, GJB2 expression levels showed significant negative correlation with the tumor infiltration levels of B cells in several tumors, especially CHOL. QUANTISEQ and TIMER database analyses showed positive correlation between GJB2 expression levels and the infiltration levels of macrophages and neutrophils in a variety of tumors. EPIC, QUANTISEQ, and TIMER database analyses showed negative correlation between GJB2 expression levels and the infiltration levels of B cells in LUSC, HNSC, ESCA, BLCA, CESC, STAD, SKCM, TGCT, CHOL, and BRCA.

EPIC database analysis showed positive correlation between GJB2 expression levels and the infiltration levels of cancer-associated fibroblasts (CAFs) in multiple tumors, especially OV and BRCA. IPS database analysis showed negative correlation between GJB2 expression levels and the levels of suppressor cells (SC), immune checkpoints (CP), and average z-scores (AZ), and positive correlation between GJB2 expression levels and the infiltration levels of effector cells (EC) in a variety of tumors. MCP algorithm results showed positive correlation between GJB2 expression levels and infiltration of the monocytic lineage, myeloid dendritic cells, neutrophils, endothelial cells, and fibroblasts, and negative correlation between GJB2 expression levels and the infiltration of T cells and the CD8^+^ T cells in several tumors. These results suggested that GJB2 expression was closely related to immune infiltration. Therefore, GJB2 may play a key role in the tumor-immune interactions.

In a few instances, the results from different algorithms were contradictory regarding the relationship between GJB2 expression levels and the immune infiltration levels. For example, the correlation between GJB2 expression levels and the B-cell infiltration levels in KIRC was positive according to the TIMER algorithm and negative according to the QUANTISEQ algorithm. Furthermore, the association between GJB2 expression levels and the infiltration levels of macrophages in DLBC was negative according to the TIMER algorithm and positive according to the EPIC algorithm. Overall, the results showed that GJB2 regulated immune infiltration in multiple tumor types.

### Functional enrichment analyses of the GJB2-related genes

**Figure 14:**
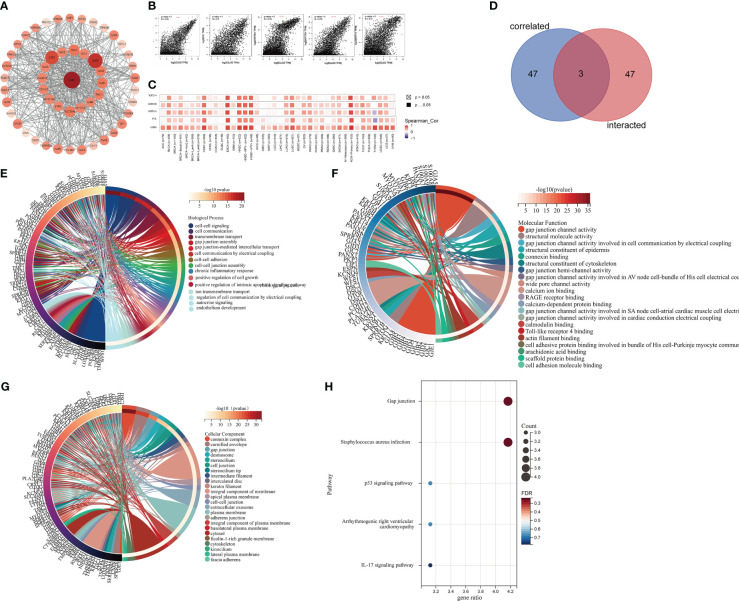
Functional enrichment analysis of GJB2-associated genes. **(A)** STRING database analysis shows identification of 50 potential GJB2-binding proteins. **(B)** GEPIA2 analysis shows identification of 50 GJB2-related genes from the TCGA database. GJB2 shows significant correlation with GJB6, KRT6A, KRT6B, KRT14, and IVL. **(C)** Heatmap shows the expression of GJB2-correlated genes in various cancer types. **(D)** Venn diagram shows the intersection between GJB2-binding genes and GJB2-related genes. **(E–G)** GO enrichment analysis results of the GJB2-binding and GJB2-related genes. **(H)** KEGG pathway analysis results of the GJB2-binding and GJB2-related genes.

The STRING database analysis identified 50 potential GJB2- binding proteins. The PPI network of GJB2 and the 50 GJB2-binding proteins is shown in **Figure 14A**. We obtained the top 50 genes associated with GJB2 expression using the GEPIA2 tool combined with all tumor expression data from TCGA.All the genes that co-express with GJB2 in the cancer tissues are shown in **Supplementary Table 1**. GJB2 expression levels showed positive correlation with the expression levels of GJB6, KRT6A, KRT6B, KRT14, and IVL (**Figure 14B**). The heat map shows positive correlation between GJB2 and the five genes (GJB6, KRT6A, KRT6B, KRT14, and IVL) in pan-cancer (**Figure 14C**).

The intersection of GJB2-binding proteins shown in the PPI network and the GJB2-coexpressing genes using the Venn diagram identified three genes, namely, *GJB5, GJB3*, and *GJB6* that were common to both the datasets (**Figure 14D**). Finally, we combined the genes related to GJB2 from both the data sets (PPI network and co-expression network) and performed functional enrichment analysis to determine the enriched KEGG pathways and GO terms. GO enrichment analysis showed that GJB2 was involved in tumorigenesis through the following pathways: 1) GJIC-dependent pathways that regulate gap junction channel activity and gap junction-mediated intercellular transport, which is the most common mode of intercellular communication and plays an important role in regulating cellular growth, and survival; GJB2 expression caused defective GJIC and resulted in abnormal cell proliferation and differentiation, thereby promoting tumorigenesis; 2) Hemichannels-dependent pathways that regulate inter cellular communication through electrical coupling, ion transmembrane transport, autocrine signaling, and participate in tumorigenesis by releasing autocrine and/or paracrine signaling molecules into the extracellular environment through the hemichannels; 3) Inflammation-related pathways that regulate neutrophil aggregation, chronic inflammatory response, positive regulation of interleukin-1 production, and facilitate oncogenesis by regulating the functions of tumor- associated inflammatory cells; 4) Other pathways including the Toll-like receptor 4 (TLR4) binding related pathway, which suppressed apoptosis and promoted tumor growth. Therefore, GJB2 may be involved in tumorigenesis by regulating the intrinsic apoptotic signaling pathway and the binding of cell adhesion molecules (**Figures 14E–G**; **Supplementary Table 2**). KEGG pathway analysis suggested that GJB2 facilitated oncogenesis by suppressing the expression of P53, a well known tumor suppressor gene (**Figure 14H**; **Supplementary Table 3**).

### Validation of GJB2 co-expression networks in PAAD

**Figure 15:**
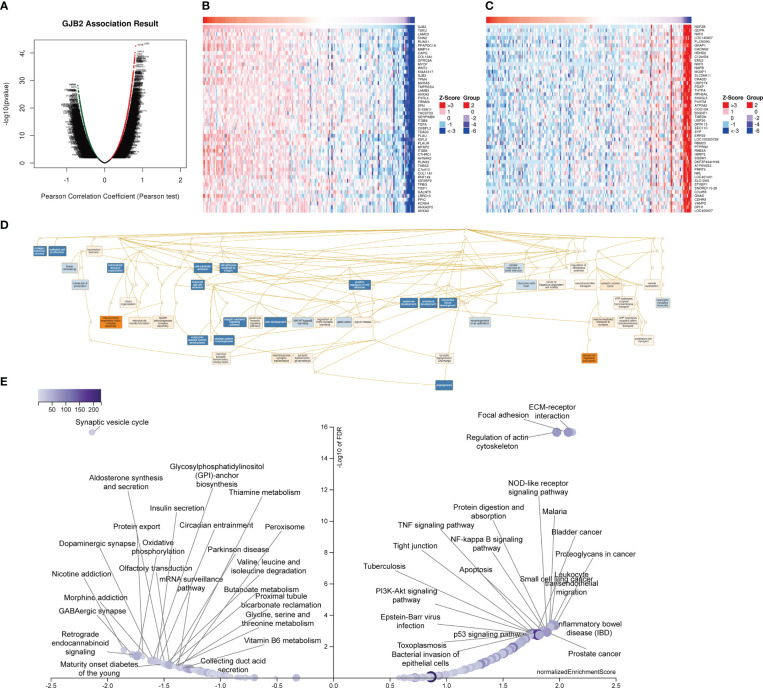
LinkedOmics database analysis of GJB2 co-expression genes in PAAD. **(A)** Pearson’s correlation test results show genes with significantly high correlation with GJB2 in the PAAD cohort. **(B, C)** Heatmaps of the top 50 genes that show **(B)** positive and **(C)** negative correlations with GJB2 in the PAAD cohort. **(D)** Directed no-loop plots for the GO analysis of GJB2-related genes in the PAAD cohort. **(E)** Volcano map shows the KEGG pathway analysis of GJB2-related genes in the PAAD cohort.

The above results confirmed that GJB2 was significantly associated with tumor prognosis and immunity. Therefore, we further verified the KEGG pathways and GO_BP terms associated with GJB2 and related genes in PAAD using the LinkedOmics database. **Figure 15A** shows the volcano map with GJB2 co-expressed genes in PAAD. **Figures 15B, C** shows the heat map with the top 50 genes that positively or negatively correlate with GJB2 in PAAD. All the GJB2 coexpressed genes in PAAD are shown in **Supplementary Table 4**. GSEA module in the LinkedOmics database was used to determine the most enriched GO terms (biological process) and the KEGG pathways in relation with the GJB2 co-expressed genes in PAAD. GO analysis showed that GJB2 was associated with integrin-mediated signaling pathways, epithelial cell proliferation, positive regulation of cell adhesion, NIK/NF-kappaB signaling, ATP hydrolysis coupled cation transmembrane transport, calcium ion- regulated exocytosis, and regulation of membrane potential (**Figure 15D**).

Integrins primarily mediate intercellular recognition and adhesion, and may be closely related with the cellular adhesion characteristics of the pancreatic adenocarcinoma cells. GJB2 facilitated pancreatic cancer development by releasing ATP through the hemichannels, thereby promoting inflammation by activating the leukocytes. Furthermore, these results also suggested that GJB2 was involved in the initiation and development of PAAD by modulating the activities of the gap junctions and hemichannels. KEGG pathway analysis showed that the GJB2 co-expressed genes were enriched in pathways such as apoptotic signaling pathway, NOD-like receptor signaling pathway, p53 signaling pathway, PI3K-Akt signaling pathway, proteoglycans in cancer, TNF signaling pathway, NF-kappa B signaling pathway, ECM-receptor interactions, and oxidative phosphorylation (**Figure 15E**). These data demonstrated that GJB2 regulated the development of pancreatic cancer by inhibiting cellular apoptosis, promoting cellular proliferation, and altering cellular differentiation through the enriched KEGG pathways.

## Discussion

Connexins are trans membrane proteins that assemble to form connexons or hemichannels, which then dock with the hemichannels of the adjacent cells to form the intercellular gap junction (GJ) channels. In 1966, Loewenstein and Kanno (18) conducted a seminal *ex vivo* study that demonstrated loss of electrical coupling in rat liver tumors and suggested a tumor suppressor role for the GJIC, which are formed from the connexins. Later, other studies confirmed the tumor suppressive function of the connexins (19–21). Subsequent studies have shown that the role of connexins or gap junctions in cancer is complex, and their function varies and is dependent on the cell type and the cancer stages. For example, the migration potential of various types of cancer cells is reduced by increased GJIC; however, high expression of connexins has been reported at the metastatic sites in glioma and colorectal cancer (22). Furthermore, high Cx26 expression is associated with poor prognosis in the renal, pancreatic, and lung cancers (23). This suggested that Cx26 played a significant role in tumorigenesis. The role of connexin-associated GJIC in cancer has been widely reported. Furthermore, there is a growing interest regarding the role of hemichannels in cancer. Several studies have reported that aberrant activation of intracellular pathways and autocrine/paracrine signaling through the hemichannels altered tumor cell proliferation and disease progression through the transmembrane exchange of signaling molecules (24–26). Paracrine signaling between tumor cells and stromal cells through the hemichannels played a significant role in tumor growth and progression (20).

It is not clear if the pathogenic role of GJB2 in different tumors is through similar or diverse molecular mechanisms. Furthermore, the relationship between GJB2 expression and various clinicopathological parameters in the pan-cancer datasets has not been studied. Therefore, we first comprehensively analyzed the GJB2 gene expression in the pan- cancer datasets from various databases. We then investigated the prognostic role of GJB2 and the underlying molecular mechanisms in different cancers by analyzing the correlations between GJB2 gene expression and the status of survival outcomes, GJB2 gene alterations, tumor immune infiltration, and related cellular pathways.

First, analysis of clinical data in the TIMER, GEPIA, and Sangerbox databases showed that GJB2 was highly expressed in 14 cancer types, including BRCA, CESC, LUAD, and STAD. This was in accordance with previous findings (6, 10, 27, 28). The HPA database analysis showed that GJB2 protein expression was significantly increased in the tumortissues compared with the corresponding normal tissues in patients with BRCA, LUAD, BLCA, UCEC, STAD, COAD, and LUSC. These results demonstrated that GJB2 played a critical role in oncogenesis. Then, we investigated the prognostic role of GJB2 in various tumors. Our results were in concordance with previous studies, which showed that high GJB2 expression was associated with worse prognosis outcomes in patients with PAAD (29), ECSA (30), and LUAD (31). Kaplan–Meier survival curve analysis showed that low expression of GJB2 correlated with better prognosis outcomes in patients with BLCA. Li et al. (32) reported that decreased Cx26 expression was associated with the progression of bladder cancer. This was contradictory to our findings. Therefore, further high-quality clinical studies are needed to confirm our findings. Previous studies have also reported contradictory findings regarding the correlation between GJB2 expression and the prognosis of gastric cancer patients. Liu et al. (10) showed that high GJB2 expression was a favorable prognostic marker for intestinal gastric cancer. However, Kim et al. (33) showed that the overexpression of Cx26 was a biomarker for poor prognosis in patients with intestinal gastric cancer. In our study, GEPIA and KM plotter database analysis showed that high GJB2 expression was associated with good prognosis outcomes in patients with gastric cancer. In the future, comprehensive clinical studies are required to further validate the correlation between gastric cancer prognosis and GJB2 expression.Our data also showed that high expression of GJB2 was associated with worse prognosis outcomes in patients with CESC, KIRC, OV, GBM, and SARC. These data demonstrated that GJB2 was a promising prognostic bio marker in pan-cancer.

Our study demonstrated that the expression levels of GJB2 were significantly associated with the clincial stages, grades, gender, and age of patients with various tumors. This suggested that GJB2 may be of great significance in guiding the clinical treatment of cancer patients belonging to different ages, genders, and tumor pathological stages. In conclusion, our study demonstrated that GJB2 played an important role in tumor progression and was a potential prognostic predictor.

In this era of precision mediciine, several studies have shown that TMB, MSI, and neoantigens are promising tumor immunity-related biomarkers for guiding immunotherapy (16, 34–36). The immune checkpoint (ICP) genes are associated with the tumor inflitration of immune cells and immunotherapy response (37). Hence, we studied the association between GJB2 expression levels and the status of TMB, MSI, neoantigens, and ICP in human cancers to determine the potential of GJB2 as a biomarker of immunotherapy response. Our findings demonstrated that GJB2 was linked to multiple immune checkpoint genes in most tumors. Furthermore, GJB2 expression correlated with several immune checkpoint genes in KICH and KIRC. This suggested that GJB2 was a potential therapeutic target for future anti-cancer immunotherapy. TMB is a predictive biomarker for accurately predicting the response of individual cancer patients for immunotherapy. TMB is also used to predict the prognosis of patient undergoing various anti-cancer treatments including immunotherapy (38). Several clinical studies have demonstrated that cancer patients with high TMB show enhanced response to treatment with immune checkpoint inhibitors such as anti-CTLA-4 therapy for melanoma (36), anti-PD-L1 therapy for uroepithelial cancer (39), and anti-PD-1 therapy for non-small cell lung cancer and colorectal cancer (40). MSI causes tumor-related gene abnormalities due to replication errors resulting in code-shifting mutations, which in turn induce cancer development. MSI is an essential clinical biomarker for immunotherapy (35). Neoantigens are abnormal proteins that are produced because of genetic mutations in the cancer cells and are not present in the normal cells. Therefore, neoantigens can activate the immune system after recognition by the immune cells and have been established as one of the biomarkers for tumor immunotherapy (41). Our study demonstrated that GJB2 expression was positively correlated with TMD in patients with LUAD, COAD, COADREAD, KIPAN, KIRP, and STAD. Furthermore, GJB2 expression was positively correlated with MSI in patients with COADREAD and STAD. Therefore, we hypothesized that patients with high GJB2 expression levels as well as high TMB and MSI may show better prognosis after immunotherapy in those cancers where the GJB2 expression levels demonstrate positive correlation with TMB and MSI.

The TME is composed of tumor cells, stromal cells such as the fibroblasts, multiple types of immune cells such as T lymphocytes, NK cells, macrophages and dendritic cells, and the extracellular matrix. The TME plays a crucial role in the tumor progression and treatment response. The immune cells and stromal cells represent two main types of non-tumor components with immense clinical value for the diagnostic and prognostic assessment of tumors. In majority of the human cancer types, GJB2 showed positive correlation with immune scores, stromal scores, and ESTIMATE scores for the TME. This implied that GJB2 expression levels were associated with TME, especially tumor infiltration of immune cells. Furthermore, the status of tumor infiltration of the immune cells is a critical parameter that is associated with the clinical response to immunotherapy (42, 43). Our results showed that GJB2 was involved in the tumor infiltration of immune cells in multiple cancer types. Furthermore, GJB2 expression levels showed positive correlation with the ESTIMATE scores in several cancer types. ESTIMATE score is negatively correlated with tumor purity (44). Low tumor purity is associated with advanced cancer stage and worse prognosis (45). Furthermore, our data showed positive correlation between GJB2 expression levels and the infiltration status of multiple types of immune cells such as cancer associated fibroblasts (CAF), macrophages, myeloid dendritic cells, DCs, neutrophils, monocytes, and endothelial cells in most tumors. Cancer- associated fibroblasts showed positive correlation with GJB2 expression in most tumors, with the strongest positive correlation in ovarian cancer. GJB2 expression also showed negative correlation with the level of B-cell infiltration in ESCA, BLCA, CESC, STAD, SKCM, TGCT, and others. These results suggested that GJB2 was strongly associated with the TME in most human cancers.

CAFs play multiple roles in tumor microenvironment. They inhibit the function of immune cells by secreting various cytokines or metabolites that promote tumor growth, invasion, and metastasis. Furthermore, CAFs play a significant role in remodeling the extra-cellular matrix, thereby reducing the effectiveness of tumor treatment by creating a barrier for the deep penetration of drugs and immune cells into the tumor tissues (46). This suggests that tumor suppression can be reversed by modulating the CAFs or overcoming their barrier effect and can be novel strategy for tumor therapy. Our results demonstrated that GJB2 was involved in immunomodulation and tumor infiltration of immune cells in multiple cancers. Therefore, GJB2 is a potential target for immunomodulation in tumor therapy that can impact tumor growth, proliferation, and progression.

Our study also perfromed function enrichment analysis to determine the biological functions of GJB2. The results of functional enrichment analysis in pan-cancer and PAAD showed that GJB2 modulated cancer progression through the p53 signaling pathway, apoptotic signaling pathway, TNF signaling pathway, PI3K-Akt signaling pathway, and others. These results were in concordance with previously published data (28, 29, 47). P53 is a well established tumor suppressor gene that regulates cell cycle and apoptosis. Nomura et al. (48) reported that Cx26 suppressed colorectal cancer by inhibiting P53 expression. Cancer progression is determined by the balance between pro-apoptotic and anti-apoptotic proteins, the mutation of cIAP, and activation of NF-κB transcriptional activity mediated by c-FLIP, all of which promote resistance of cancer cells resistant to apoptotic stimuli (49). PI3K-Akt signaling pathway is commonly dysregulated in several human cancers. This is caused by mutations in the proteins involved in this pathway through gain of function or loss of function mutations. Aberrant regulation of the PI3K-Akt signaling pathway promotes cellular transformation and also regulates tumor cell proliferation, survival, and invasiveness (50). We investigated the potential biological functions of GJB2 through the GO analysis. GJB2 expression was associated with biological processes such as gap junction-mediated intercellular transport, positive regulation of interleukin-1 production, cell adhesion molecule binding, positive regulation of intrinsic apoptotic signaling pathway, regulation of cell growth, negative regulation of mRNA catabolic process, RAGE receptor binding, Toll-like receptor 4 binding and morphogenesis of an epithelium.

Previous studies have also shown that GJIC mediated by Cx26 promotes tumorigenesis by regulating cell proliferation and differentiation and facilitates cancer cell migration by reducing cell adhesion (51). The presence of DAMPs in cells stimulates the secretion of inflammatory cytokines induced by the toll-like receptors (TLR), thereby inducing chronic inflammation in the tumor microenvironment. Previous studies have shown that the hemichannels formed by GJB2 are closely associated with tumorigenesis (26, 52, 53), which agrees with the findings of our study. Our study showed that GJB2 was involved in tumor cell proliferation and migration mainly through regulation of cell communication by electrical coupling, ion transmembrane transport, and autocrine signaling. Our results also suggested that GJB2 regulated tumorigenesis through inflammation-related pathways, which was in line with previous findings (54, 55). Hemichannels of connexins promote inflammatory responses. Tumor cells produce cytokines that attract tumor-associated inflammatory cells such as neutrophils (TANs) and macrophages (TAMs) to the tumor site. TANs and TAMs promote tumor growth by secreting growth factors that also induce tumor angiogenesis. These changes promote tumor progression, inhibit apoptosis of tumor cells, and induce tumor resistance to immune responses (56). In conclusion, these data suggested that GJB2 modulated tumorigenesis through diverse mechanisms.

Overall, our findings demonstrated that GJB2 was an independent prognostic factor for manifold cancers Furthermore, GJB2 expression correlated with TMB, MSI, ICP, neoantigens, and tumor infiltration of immune cells in diverse cancer types. The impact of GJB2 on tumor immunity also varies depending on the tumor type. As a result, we hypothesized that GJB2 was not only a promising prognostic factor for multiple cancer types but also a potential target for immunotherapy. Our data provides the basis for exploring the clinical applications of GJB2-targeted cancer immunotherapy in the future preclinical and clinical studies as well as further exploring the biological role of GJB2.

Our study has some limitations. First, we analyzed clinical data from different databases. There were a few differences in the data across databases that could have resulted in bias. Secondl our data regarding the biological function of GJB2 needs to be confirmed through *in vivo* and *in vitro* experiments. Third, we concluded that GJB2 expression was strongly associated with immune cell infiltration and prognosis of human cancers. However, we did not provide direct evidence for the role of GJB2 in tumor immune infiltration and its relationship with prognosis. Finally, none of the anti-GJB2 targeting drugs have been tested so far in clinical trials. Therefore, currently, the potential immunotherapeutic effects of anti-GJB2 treatment is speculative. In the future, there is a need to develop and test anti-tumor immunotherapeutic agents targeting GJB2. Moreover, future prospective studies with larger sample sizes are needed to further validate the clinical value of GJB2 in pan-cancer.

## Author contributions

YJ and EL conceived and designed this study. YJ and BG performed the bioinformatics analyses and visualization. WZ, FW and YZ collected the data and performed the statistical analysis. YJ and QZ wrote the original draft. EL and YZ revised the manuscripts. All authors contributed to the article and approved the submitted version.

## Supplementary material

Supplementary Figure 3 Correlation analysis between GJB2 expression and stromal scores in pan-cancer using the ESTIMATE algorithm.

Supplementary Figure 4 Correlation analysis between GJB2 expression and immune scores in pan-cancer using the ESTIMATE algorithm.

Supplementary Figure 5 CIBERSORT analysis results show that GJB2 expression levels are significantly correlated with the infiltration levels of various immune cells. ∗*P* < 0.05.

Supplementary Figure 6 MCPCOUNTER analysis results show significant correlation between GJB2 expression levels and the infiltration levels of various immune cells. ∗*P* < 0.05, ∗∗*P* < 0.01, and ∗∗∗*P* < 0.001.

Supplementary Figure 7 TIMER database analysis results show significant correlation between GJB2 expression levels and the infiltration levels of various immune cells. ∗*P* < 0.05, ∗∗*P* < 0.01, and ∗∗∗*P* < 0.001.

## Glossary

**Table:**

ACC	adrenocortical carcinoma
BLCA	bladder urothelial carcinoma
BRCA	breast carcinoma
CESC	cervical squamous cell carcinoma and endocervical adenocarcinoma
CHOL	cholangiocarcinoma
COAD	colon adenocarcinoma
DLBCL	diffuse large B-cell lymphoma
ESCA	esophageal carcinoma
GBM	glioblastoma multiforme
HNSC	head and neck squamous cell carcinoma
KICH	kidney chromophobe
KIRC	kidney renal clear cell carcinoma
KIRP	kidney renal papillary cell carcinoma
LAML	acute myeloid leukemia
LIHC	liver hepatocellular carcinoma
LUAD	lung adenocarcinoma
LUSC	lung squamous cell carcinoma
MESO	mesothelioma
OV	ovarian serous cystadenocarcinoma
PAAD	pancreatic adenocarcinoma
PCPG	pheochromocytoma and paraganglioma
PRAD	prostate adenocarcinoma
READ	rectal adenocarcinoma
SARC	sarcoma
SKCM	skin cutaneous melanoma
STAD	stomach adenocarcinoma
TGCT	testicular germ cell tumors
THCA	thyroid carcinoma
THYM	thymoma
UCEC	uterine corpus endometrial carcinoma
	
	
STES	Stomach and Esophageal carcinoma
COADREAD	Colon adenocarcinoma/Rectum adenocarcinoma Esophageal carcinoma
GBMLGG	Glioma
KIPAN	Pan-kidney cohort
HPA	Human Protein Atlas
GEPIA2	Gene Expression Profiling Interactive Analysis 2
GEO	Gene Expression Omnibus
TCGA	The cancer genome atlas
TME	Tumor microenvironment
CAN	copy number alteration
OS	overall survival
	
DFS	disease-free survival
TMB	tumor mutation burden. ICP, immune checkpoint
MSI	microsatellite instability
NEO	Neoantigen
KEGG	Kyoto Encyclopedia of Genes and Genomes
GO	Gene Ontology.

## References

[1] Sung H Ferlay J Siegel RL Laversanne M Soerjomataram I Jemal A. Global cancer statistics 2020: GLOBOCAN estimates of incidence and mortality worldwide for 36 cancers in 185 countries. CA: Cancer J Clin (2021) 71(3):209–49. doi: 10.3322/caac.21660 33538338

[2] Hinshaw DC Shevde LA. The tumor microenvironment innately modulates cancer progression. Cancer Res (2019) 79(18):4557–66. doi: 10.1158/0008-5472.CAN-18-3962 PMC6744958 31350295

[3] Siegel RL Miller KD Fuchs HE Jemal A. Cancer statistics, 2022. CA: Cancer J Clin (2022) 72(1):7–33. doi: 10.3322/caac.21708 35020204

[4] Omori Y Duflot-Dancer A Mesnil M Yamasaki H. Role of connexin (gap junction) genes in cell growth control: approach with site-directed mutagenesis and dominant-negative effects. Toxicol Lett (1998) 96-97:105–10. doi: 10.1016/S0378-4274(98)00056-3 9820654

[5] Wei CJ Xu X Lo CW. Connexins and cell signaling in development and disease. Annu Rev Cell Dev Biol (2004) 20:811–38. doi: 10.1146/annurev.cellbio.19.111301.144309 15473861

[6] Naoi Y Miyoshi Y Taguchi T Kim SJ Arai T Tamaki Y. Connexin26 expression is associated with lymphatic vessel invasion and poor prognosis in human breast cancer. Breast Cancer Res Treat (2007) 106(1):11–7. doi: 10.1007/s10549-006-9465-8 17203385

[7] Ezumi K Yamamoto H Murata K Higashiyama M Damdinsuren B Nakamura Y. et al: aberrant expression of connexin 26 is associated with lung metastasis of colorectal cancer. Clin Cancer research: an Off J Am Assoc Cancer Res (2008) 14(3):677–84. doi: 10.1158/1078-0432.CCR-07-1184 18245526

[8] Yang J Qin G Luo M Chen J Zhang Q Li L. Reciprocal positive regulation between Cx26 and PI3K/Akt pathway confers acquired gefitinib resistance in NSCLC cells *via* GJIC-independent induction of EMT. Cell Death Dis (2015) 6(7):e1829. doi: 10.1038/cddis.2015.197 26203858 PMC4650742

[9] Van Campenhout R Gomes AR De Groof TWM Muyldermans S Devoogdt N Vinken M. Mechanisms underlying connexin hemichannel activation in disease. Int J Mol Sci (2021) 22(7):1–14. doi: 10.3390/ijms22073503 PMC8036530 33800706

[10] Liu X Furuya T Li D Xu J Cao X Li Q. Connexin 26 expression correlates with less aggressive phenotype of intestinal type-gastric carcinomas. Int J Mol Med (2010) 25(5):709–16. doi: 10.3892/ijmm_00000395 20372813

[11] Tang Z Kang B Li C Chen T Zhang Z. GEPIA2: an enhanced web server for large-scale expression profiling and interactive analysis. Nucleic Acids Res (2019) 47(W1):W556–w560. doi: 10.1093/nar/gkz430 31114875 PMC6602440

[12] Cerami E Gao J Dogrusoz U Gross BE Sumer SO Aksoy BA. The cBio cancer genomics portal: an open platform for exploring multidimensional cancer genomics data. Cancer Discovery (2012) 2(5):401–4. doi: 10.1158/2159-8290.CD-12-0095 PMC3956037 22588877

[13] Hu J Yu A Othmane B Qiu D Li H Li C. Siglec15 shapes a non-inflamed tumor microenvironment and predicts the molecular subtype in bladder cancer. Theranostics (2021) 11(7):3089–108. doi: 10.7150/thno.53649 PMC7847675 33537076

[14] Darvin P Toor SM Sasidharan Nair V Elkord E. Immune checkpoint inhibitors: recent progress and potential biomarkers. Exp Mol Med (2018) 50(12):1–11. doi: 10.1038/s12276-018-0191-1 PMC6292890 30546008

[15] Schumacher TN Schreiber RD. Neoantigens in cancer immunotherapy. Sci (New York NY) (2015) 348(6230):69–74. doi: 10.1126/science.aaa4971 25838375

[16] Peng M Mo Y Wang Y Wu P Zhang Y Xiong F. Neoantigen vaccine: an emerging tumor immunotherapy. Mol Cancer (2019) 18(1):128. doi: 10.1186/s12943-019-1055-6 31443694 PMC6708248

[17] Yarchoan M Hopkins A Jaffee EM. Tumor mutational burden and response rate to PD-1 inhibition. New Engl J Med (2017) 377(25):2500–1. doi: 10.1056/NEJMc1713444 PMC6549688 29262275

[18] Loewenstein WR Kanno Y. Intercellular communication and the control of tissue growth: lack of communication between cancer cells. Nature (1966) 209(5029):1248–9. doi: 10.1038/2091248a0 5956321

[19] Mesnil M Crespin S Avanzo JL Zaidan-Dagli ML. Defective gap junctional intercellular communication in the carcinogenic process. Biochim Biophys Acta (2005) 1719(1-2):125–45. doi: 10.1016/j.bbamem.2005.11.004 16359943

[20] Aasen T Leithe E Graham SV Kameritsch P Mayán MD Mesnil M. Connexins in cancer: bridging the gap to the clinic. Oncogene (2019) 38(23):4429–51. doi: 10.1038/s41388-019-0741-6 PMC6555763 30814684

[21] Alaga KC Crawford M Dagnino L Laird DW. Aberrant Cx43 expression and mislocalization in metastatic human melanomas. J Cancer (2017) 8(7):1123–8. doi: 10.7150/jca.18569 PMC5463425 28607585

[22] Strale PO Clarhaut J Lamiche C Cronier L Mesnil M Defamie N. Down-regulation of Connexin43 expression reveals the involvement of caveolin-1 containing lipid rafts in human U251 glioblastoma cell invasion. Mol carcinogenesis (2012) 51(11):845–60. doi: 10.1002/mc.20853 21882259

[23] Unal YC Yavuz B Ozcivici E Mese G. The role of connexins in breast cancer: from misregulated cell communication to aberrant intracellular signaling. Tissue barriers (2022) 10(1):1962698. doi: 10.1080/21688370.2021.1962698 34355641 PMC8794248

[24] Stout CE Costantin JL Naus CC Charles AC. Intercellular calcium signaling in astrocytes *via* ATP release through connexin hemichannels. J Biol Chem (2002) 277(12):10482–8. doi: 10.1074/jbc.M109902200 11790776

[25] Gossman DG Zhao HB. Hemichannel-mediated inositol 1,4,5-trisphosphate (IP3) release in the cochlea: a novel mechanism of IP3 intercellular signaling. Cell communication adhesion (2008) 15(4):305–15. doi: 10.1080/15419060802357217 PMC5543712 18979296

[26] Schalper KA Carvajal-Hausdorf D Oyarzo MP. Possible role of hemichannels in cancer. Front Physiol (2014) 5:237. doi: 10.3389/fphys.2014.00237 25018732 PMC4073485

[27] Meng S Liu Y Wang X Wu X Xie W Kang X. The prognostic value and biological significance of gap junction beta protein 2 (GJB2 or Cx26) in cervical cancer. Front Oncol (2022) 12:907960. doi: 10.3389/fonc.2022.907960 35936685 PMC9355537

[28] Tang Y Zhang YJ Wu ZH. High GJB2 mRNA expression and its prognostic significance in lung adenocarcinoma: a study based on the TCGA database. Medicine (2020) 99(14):e19054. doi: 10.1097/MD.0000000000019054 32243356 PMC7220691

[29] Zhu T Gao YF Chen YX Wang ZB Yin JY Mao XY. Genome-scale analysis identifies GJB2 and ERO1LB as prognosis markers in patients with pancreatic cancer. Oncotarget (2017) 8(13):21281–9. doi: 10.18632/oncotarget.15068 PMC5400583 28177904

[30] Inose T Kato H Kimura H Faried A Tanaka N Sakai M. Correlation between connexin 26 expression and poor prognosis of esophageal squamous cell carcinoma. Ann Surg Oncol (2009) 16(6):1704–10. doi: 10.1245/s10434-009-0443-3 19326169

[31] Lu A Shi Y Liu Y Lin J Zhang H Guo Y. Integrative analyses identified ion channel genes GJB2 and SCNN1B as prognostic biomarkers and therapeutic targets for lung adenocarcinoma. Lung Cancer (Amsterdam Netherlands) (2021) 158:29–39. doi: 10.1016/j.lungcan.2021.06.001 34111567

[32] Li X Su Y Pan J Zhou Z Song B Xiong E. Connexin 26 is down-regulated by KDM5B in the progression of bladder cancer. Int J Mol Sci (2013) 14(4):7866–79. doi: 10.3390/ijms14047866 PMC3645721 23579952

[33] Kim EY Jun KH Yim K. The roles of connexin 26, 32, and 43 as prognostic factors for gastric cancer. Anticancer Res (2020) 40(8):4537–45. doi: 10.21873/anticanres.14459 32727784

[34] Samstein RM Lee CH Shoushtari AN Hellmann MD Shen R Janjigian YY. Tumor mutational load predicts survival after immunotherapy across multiple cancer types. Nat Genet (2019) 51(2):202–6. doi: 10.1038/s41588-018-0312-8 PMC6365097 30643254

[35] Boland CR Goel A. Microsatellite instability in colorectal cancer. Gastroenterology (2010) 138(6):2073–2087.e2073. doi: 10.1053/j.gastro.2009.12.064 20420947 PMC3037515

[36] Snyder A Makarov V Merghoub T Yuan J Zaretsky JM Desrichard A. Genetic basis for clinical response to CTLA-4 blockade in melanoma. New Engl J Med (2014) 371(23):2189–99. doi: 10.1056/NEJMoa1406498 PMC4315319 25409260

[37] Topalian SL Drake CG Pardoll DM. Immune checkpoint blockade: a common denominator approach to cancer therapy. Cancer Cell (2015) 27(4):450–61. doi: 10.1016/j.ccell.2015.03.001 PMC4400238 25858804

[38] Chan TA Yarchoan M Jaffee E Swanton C Quezada SA Stenzinger A. Development of tumor mutation burden as an immunotherapy biomarker: utility for the oncology clinic. Ann Oncol (2019) 30(1):44–56. doi: 10.1093/annonc/mdy495 30395155 PMC6336005

[39] Rosenberg JE Hoffman-Censits J Powles T van der Heijden MS Balar AV Necchi A. et al: atezolizumab in patients with locally advanced and metastatic urothelial carcinoma who have progressed following treatment with platinum-based chemotherapy: a single-arm, multicentre, phase 2 trial. Lancet (London England) (2016) 387(10031):1909–20. doi: 10.1016/S0140-6736(16)00561-4 PMC5480242 26952546

[40] Rizvi NA Hellmann MD Snyder A Kvistborg P Makarov V Havel JJ. Cancer immunology. mutational landscape determines sensitivity to PD-1 blockade in non-small cell lung cancer. Sci (New York NY) (2015) 348(6230):124–8. doi: 10.1126/science.aaa1348 PMC4993154 25765070

[41] Xie N Shen G Gao W Huang Z Huang C Fu L. Neoantigens: promising targets for cancer therapy. Signal Transduction Targeted Ther (2023) 8(1):9. doi: 10.1038/s41392-022-01270-x PMC9816309 36604431

[42] Wei G Zhang H Zhao H Wang J Wu N Li L. Emerging immune checkpoints in the tumor microenvironment: implications for cancer immunotherapy. Cancer Lett (2021) 511:68–76. doi: 10.1016/j.canlet.2021.04.021 33957184

[43] Roma-Rodrigues C Mendes R Baptista PV Fernandes AR. Targeting tumor microenvironment for cancer therapy. Int J Mol Sci (2019) 20(4):1–31. doi: 10.3390/ijms20040840 PMC6413095 30781344

[44] Yoshihara K Shahmoradgoli M Martínez E Vegesna R Kim H Torres-Garcia W. Inferring tumour purity and stromal and immune cell admixture from expression data. Nat Commun (2013) 4:2612. doi: 10.1038/ncomms3612 24113773 PMC3826632

[45] Aran D Sirota M Butte AJ. Systematic pan-cancer analysis of tumour purity. Nat Commun (2015) 6:8971. doi: 10.1038/ncomms9971 26634437 PMC4671203

[46] Chen Y McAndrews KM Kalluri R. Clinical and therapeutic relevance of cancer-associated fibroblasts. Nat Rev Clin Oncol (2021) 18(12):792–804. doi: 10.1038/s41571-021-00546-5 34489603 PMC8791784

[47] Yang J Qin G Luo M Chen J Zhang Q Li L. Reciprocal positive regulation between Cx26 and PI3K/Akt pathway confers acquired gefitinib resistance in NSCLC cells *via* GJIC-independent induction of EMT. Cell Death Dis (2015) 6(7):e1829–9. doi: 10.1038/cddis.2015.197 PMC4650742 26203858

[48] Nomura S Maeda K Noda E Inoue T Fukunaga S Nagahara H. Clinical significance of the expression of connexin26 in colorectal cancer. J Exp Clin Cancer research: CR (2010) 29(1):79. doi: 10.1186/1756-9966-29-79 20565955 PMC2907868

[49] Melet A Song K Bucur O Jagani Z Grassian AR Khosravi-Far R. Apoptotic pathways in tumor progression and therapy. Adv Exp Med Biol (2008) 615:47–79. doi: 10.1007/978-1-4020-6554-5_4 18437891

[50] Fresno Vara JA Casado E de Castro J Cejas P Belda-Iniesta C González-Barón M. PI3K/Akt signalling pathway and cancer. Cancer Treat Rev (2004) 30(2):193–204. doi: 10.1016/j.ctrv.2003.07.007 15023437

[51] Wu JI Wang LH. Emerging roles of gap junction proteins connexins in cancer metastasis, chemoresistance and clinical application. J Biomed Sci (2019) 26(1):8. doi: 10.1186/s12929-019-0497-x 30642339 PMC6332853

[52] Buratto D Donati V Zonta F Mammano F. Harnessing the therapeutic potential of antibodies targeting connexin hemichannels. Biochim Biophys Acta (BBA) - Mol Basis Dis (2021) 1867(4):166047. doi: 10.1016/j.bbadis.2020.166047 33418036

[53] Retamal MA Fernandez-Olivares A Stehberg J. Over-activated hemichannels: a possible therapeutic target for human diseases. Biochim Biophys Acta (BBA) - Mol Basis Dis (2021) 1867(11):166232. doi: 10.1016/j.bbadis.2021.166232 34363932

[54] Taniguchi K Karin M. NF-κB, inflammation, immunity and cancer: coming of age. Nat Rev Immunol (2018) 18(5):309–24. doi: 10.1038/nri.2017.142 29379212

[55] Casbon AJ Reynaud D Park C Khuc E Gan DD Schepers K. Invasive breast cancer reprograms early myeloid differentiation in the bone marrow to generate immunosuppressive neutrophils. Proc Natl Acad Sci United States America (2015) 112(6):E566–575. doi: 10.1073/pnas.1424927112 PMC4330753 25624500

[56] Kashani B Zandi Z Pourbagheri-Sigaroodi A Bashash D Ghaffari SH. The role of toll-like receptor 4 (TLR4) in cancer progression: a possible therapeutic target? J Cell Physiol (2021) 236(6):4121–37. doi: 10.1002/jcp.30166 33230811

