# Supplementary figures and images for: Pan-cancer analysis of the prognostic and immunological role of GJB2: a potential target for survival and immunotherapy
Source: Front Oncol. 2023 Jun 23;13:1110207. doi: 10.3389/fonc.2023.1110207 (PMC10327570; doi:10.3389/fonc.2023.1110207)

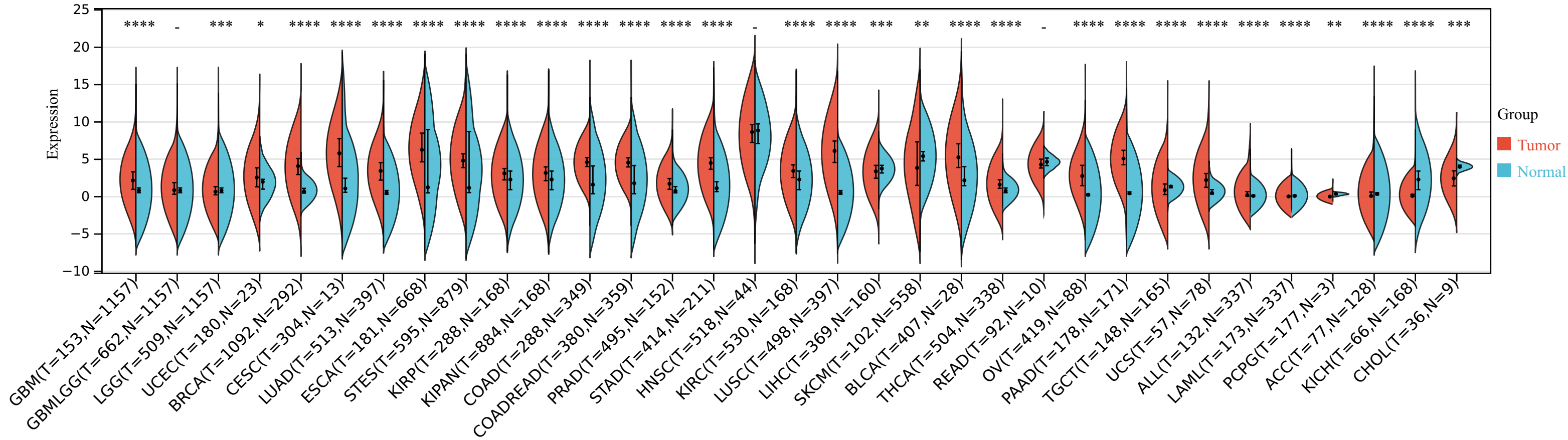

Supplement: Supplementary file 5 [file Image_1.pdf]

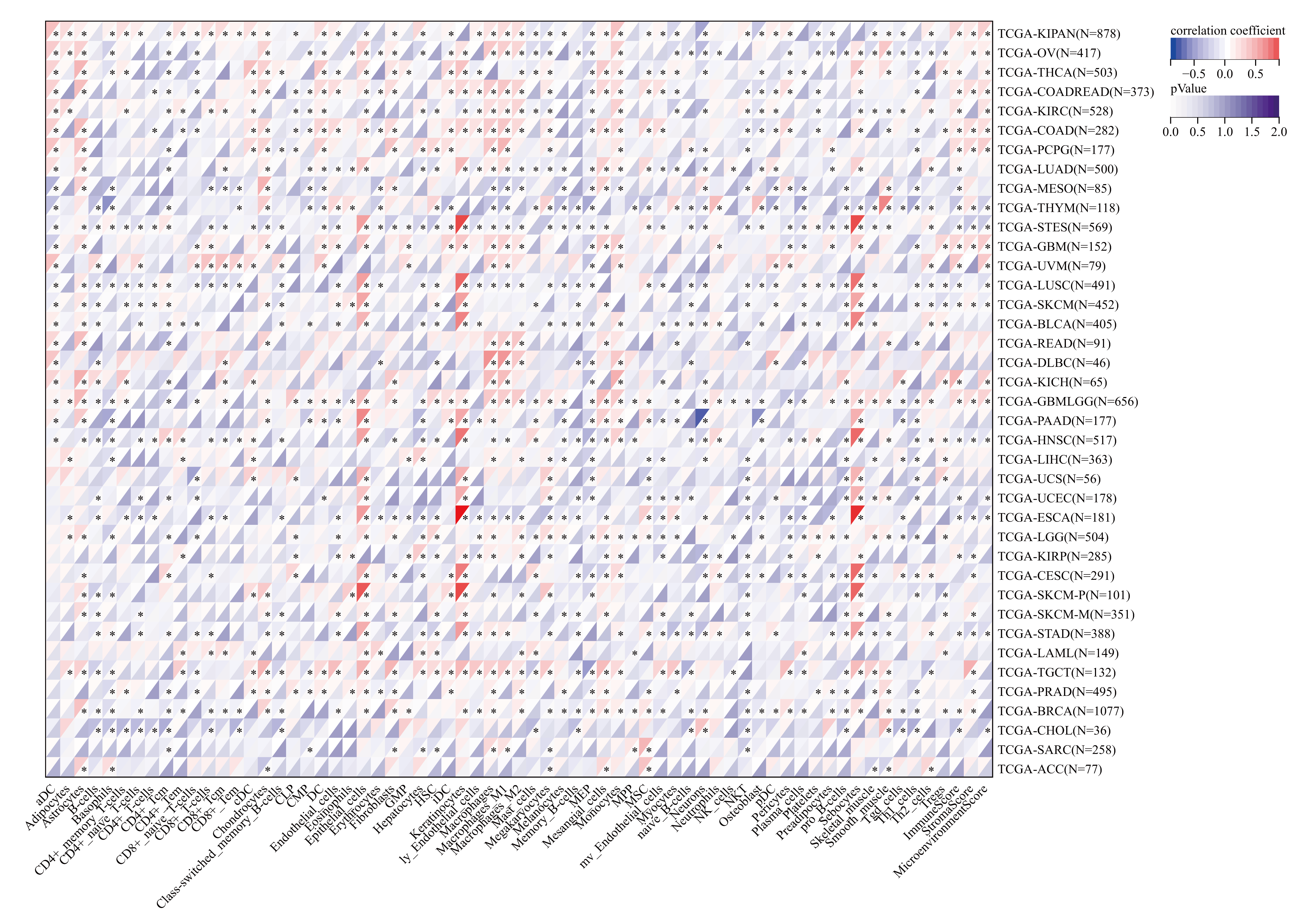

Supplement: Supplementary file 6 [file Image_2.tif]

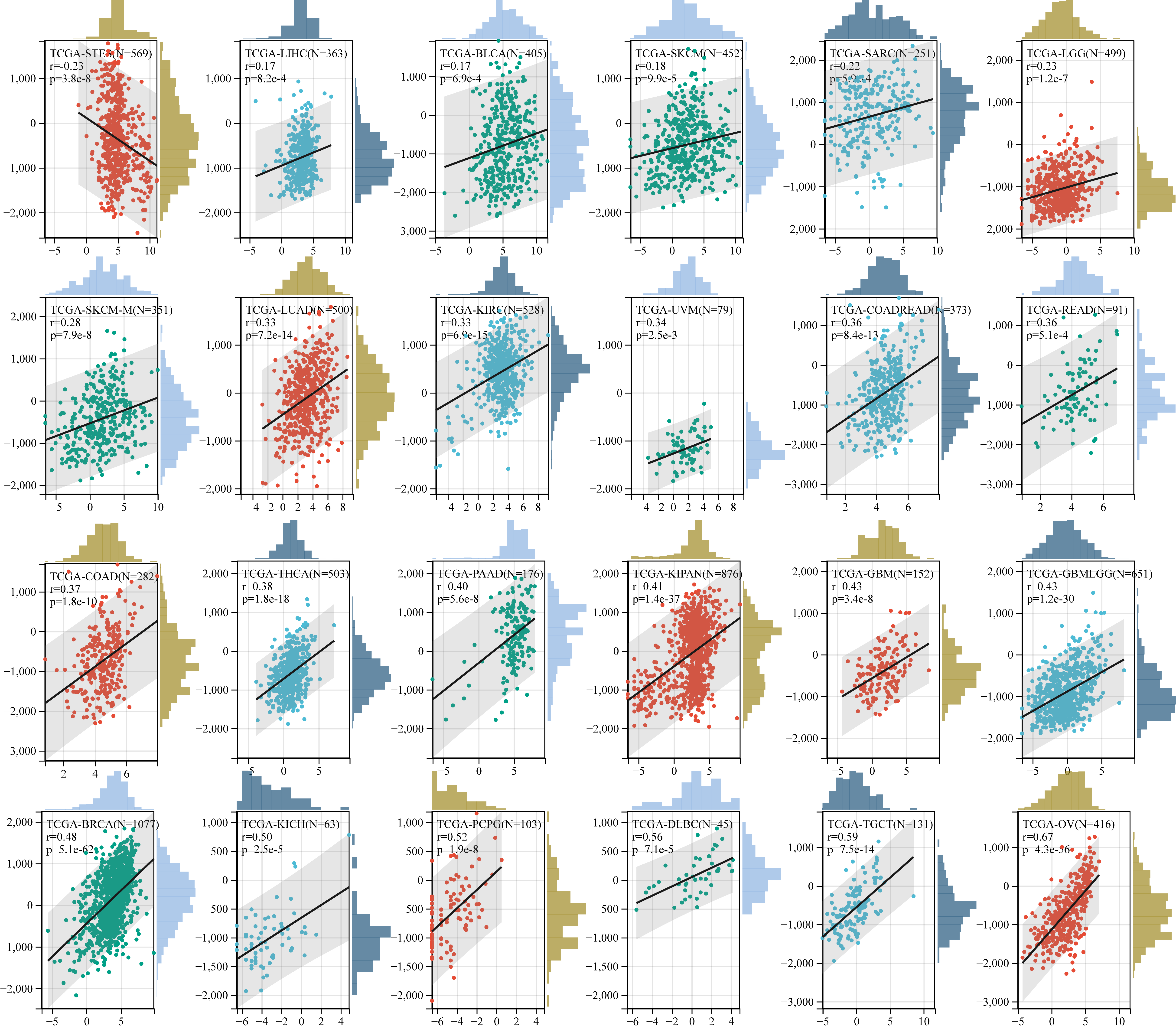

Supplement: Supplementary Figure 3 — Correlation analysis between GJB2 expression and stromal scores in pan-cancer using the ESTIMATE algorithm. [file Image_3.tif]

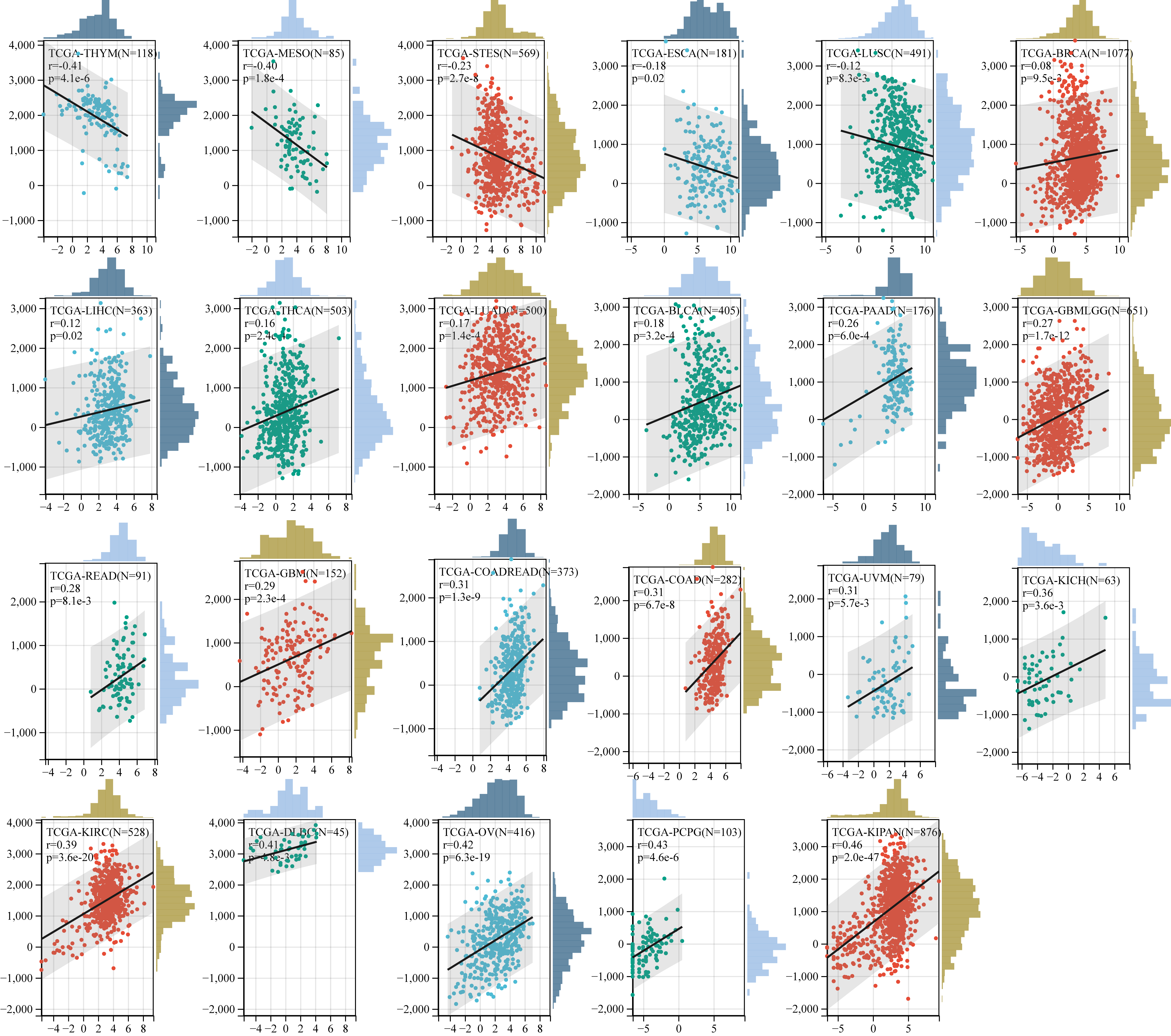

Supplement: Supplementary Figure 4 — Correlation analysis between GJB2 expression and immune scores in pan-cancer using the ESTIMATE algorithm. [file Image_4.tif]

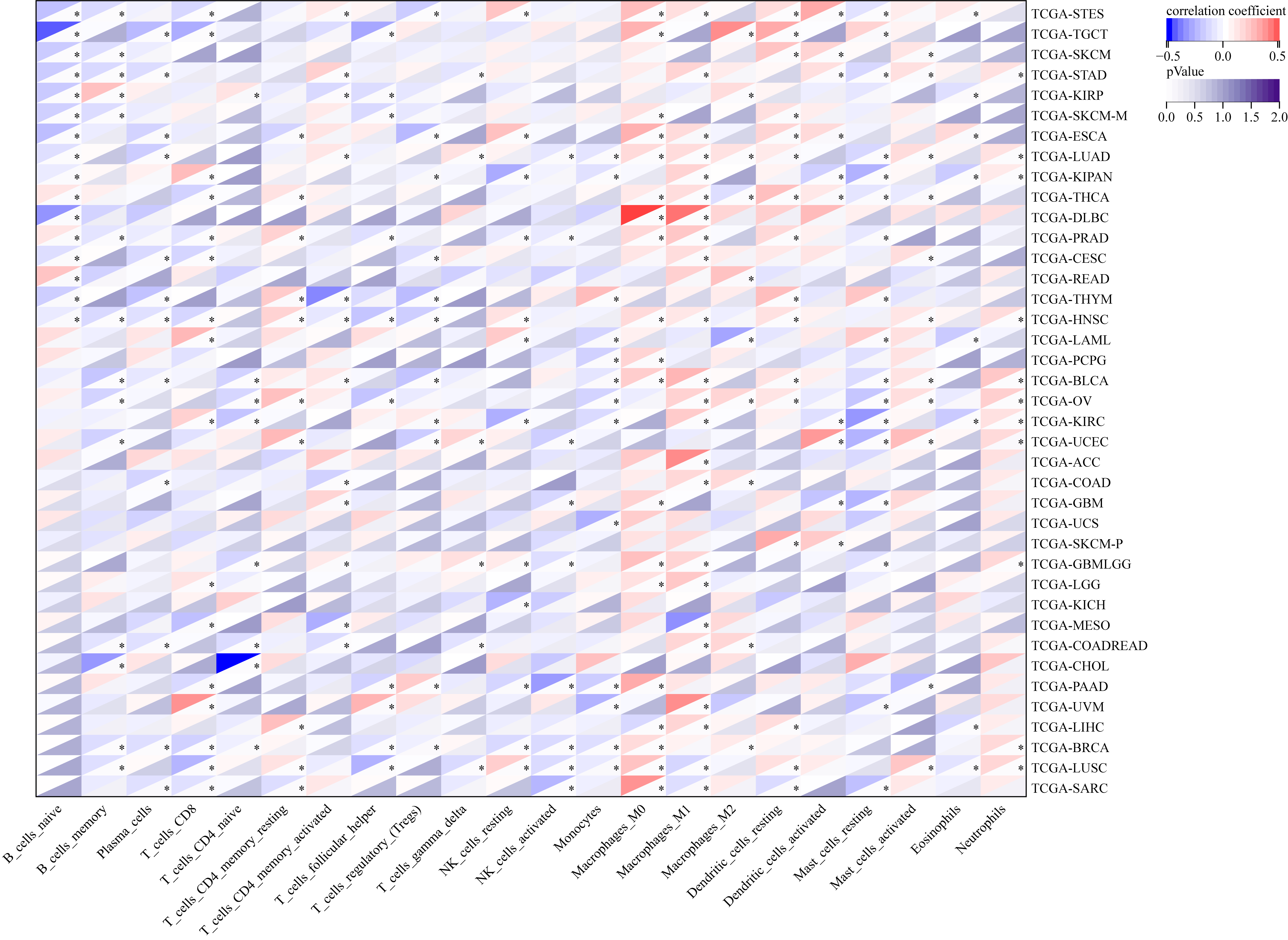

Supplement: Supplementary Figure 5 — CIBERSORT analysis results show that GJB2 expression levels are significantly correlated with the infiltration levels of various immune cells. ∗P < 0.05. [file Image_5.tif]

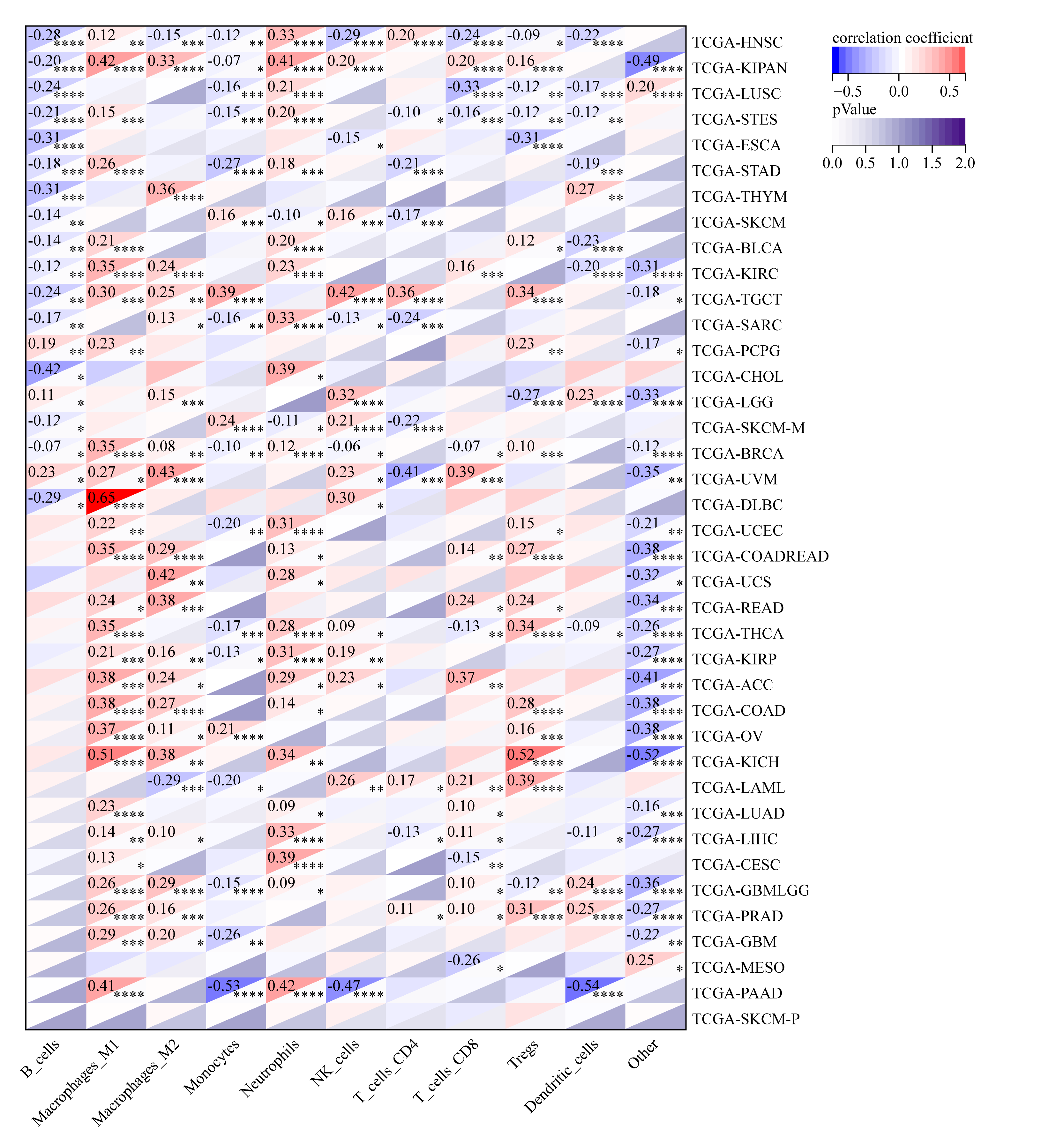

Supplement: Supplementary Figure 6 — MCPCOUNTER analysis results show significant correlation between GJB2 expression levels and the infiltration levels of various immune cells. ∗P < 0.05, ∗∗P < 0.01, and ∗∗∗P < 0.001. [file Image_6.tif]

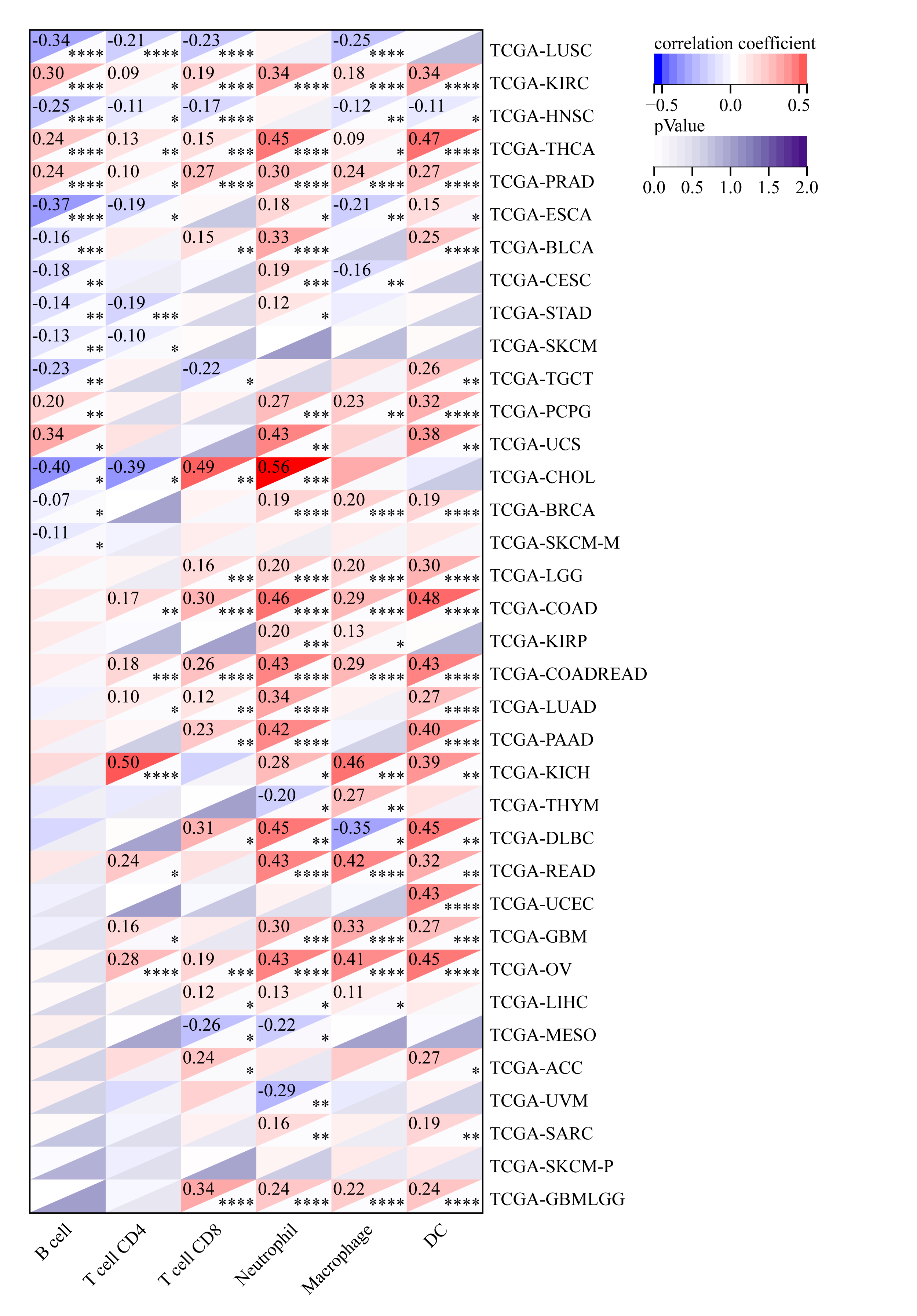

Supplement: Supplementary Figure 7 — TIMER database analysis results show significant correlation between GJB2 expression levels and the infiltration levels of various immune cells. ∗P < 0.05, ∗∗P < 0.01, and ∗∗∗P < 0.001. [file Image_7.tif]
